# Per- and polyfluoroalkyl substances (PFASs) in Swedish household dust and exposure of pet cats

**DOI:** 10.1007/s11356-021-13343-5

**Published:** 2021-03-20

**Authors:** Jana M. Weiss, Bernt Jones, Jacco Koekkoek, Anders Bignert, Marja H. Lamoree

**Affiliations:** 1 Department of Environmental Science, Stockholm University, Svante Arrheniusväg 12, 10691 Stockholm, Sweden; 2 Department of Clinical Sciences, Swedish University of Agricultural Sciences, Box 7054, 75007 Uppsala, Sweden; 3 Department Environment & Health, Vrije Universiteit, De Boelelaan 1087, 1081HV Amsterdam, The Netherlands

**Keywords:** PFAS, Dust, Cats, Serum, Thyroid, PFOS, PFOA, PAPs

## Abstract

Per- and polyfluoroalkyl substances (PFASs) are used in a wide range of products and have been found ubiquitously in our indoor environment, and there is evidence that exposure to PFAS can lead to adverse endocrine effects, such as thyroid hormone disruption. Pet cats have a high dust intake due to their grooming behavior and have been shown to be a suitable sentinel species for assessment of toddler’s exposure. Here we used paired household dust (*n*=46) and cat serum (*n*=27) samples to establish whether dust is a relevant exposure pathway to PFASs. An analytical method for PFAS analysis was optimized using a low volume of cat serum samples, combining solid-phase extraction and online sample cleanup. Dust was extracted with methanol by sonication and cleaned up by addition of active carbon. In total, 27 PFASs were analyzed by liquid chromatography/mass spectrometry analysis. The correlation between PFAS levels in dust and serum, serum lipids and thyroid hormone levels, and PFAS levels in dust between different rooms were statistically evaluated. PFOS and PFDA could be quantified in all cat serum samples (median 2300 pg/mL and 430 pg/mL, respectively), followed by PFOA (median 1100 pg/mL), quantified in 96% of the samples. The levels of 6:2 and 8:2 diPAPs were determined in 65% and 92% of the serum samples, respectively, and were an order of magnitude lower (1.4–160 pg/mL). Household dust on the other hand was dominated by 6:2 and 8:2 diPAPs, with a median of 65 ng/g dust and 49 ng/g dust, respectively. PFOS (median 13 ng/g dust) and PFOA (median 9 ng/g dust) were quantified in 93% of the dust samples. Only eight PFASs were detected (>LOD) in at least 50% of the samples of both matrices and could be paired. Significant correlations between cat serum and dust were found for PFOA (*r*_S_=0.32, *p*<0.049) and PFUnDA (*r*_S_=0.55, *p*<0.001). Significant positive correlations were found between serum total thyroxine (*r*_S_=0.11, *p*<0.05) and PFNA and between serum cholesterol and PFHpA (*r*_S_=0.46, *p*<0.01), PFUnDA (*r*_S_=0.40, *p*<0.05), PFDoDA (*r*_S_=0.44, *p*<0.01), and sum PFAS (*r*_S_=0.48, *p*<0.01). In conclusion, this study confirmed that dust is a relevant exposure pathway for the ingestion of some PFASs for cats, and the serum levels of PFASs could be of relevance for the cat’s health.

**Supplementary Information:**

The online version contains supplementary material available at 10.1007/s11356-021-13343-5.

## Introduction

It has been shown over the last decades that anthropogenic organohalogen contaminants (OHCs) may interact with hormones and act as endocrine disrupting compounds (EDCs) in wildlife and humans, leading to various diseases, such as diabetes and cancers related to the endocrine system (WHO/UNEP 2013). In addition, exposure to EDCs may lead to the onset of immune-related, thyroid-related, and neurodevelopmental disorders (WHO/UNEP 2013). Thyroid hormone disorders have gained increased attention as evidence supporting the disruption of thyroid hormone mechanisms in humans and wildlife during the sensitive embryonic and postnatal development have been repeatedly reported (WHO/UNEP 2013). There are several pathways through which organisms may be exposed to EDCs, with food being one of the major routes for OHCs. Another significant exposure route is dust ingestion, especially for toddlers and children at developing stages due to their extensive hand-to-mouth activity and low body weight. For modeling purposes, the estimated daily intakes of dust ingested are between 40 and 100 mg for children and 20–60 mg for adults (US-EPA 2017; Jones-Otazo et al. 2005).

Domestic pet cats have been used as a sentinel species for exposure to OHCs accumulated in dust in indoor home environments due to their high dust intake related to their natural grooming behavior (Ali et al. 2013; Bost et al. 2016; Chow et al. 2015; Dirtu et al. 2013; Dye et al. 2007; Guo et al. 2016; Henríquez-Hernández et al. 2017; Mizukawa et al. 2016; Mizukawa et al. 2017; Norrgran Engdahl et al. 2017; Walter et al. 2017; Wang et al. 2018). To our knowledge, there are no studies estimating the daily intake of dust for cats, although correlation between levels of OHCs in dust and cat serum has been confirmed (Norrgran Engdahl et al. 2017). In addition, cats have an increasing prevalence of developing hyperthyroidism, an endocrine disorder also increasing in humans, and associations have been found between elevated levels of certain polybrominated diphenyl ethers (PBDEs) in hyperthyroid cats compared to levels in healthy cats (Norrgran et al. 2015). Feline hyperthyroidism is manifested as a disturbed upregulation of the thyroid hormone (TH), thyroxine (T4) concentration, and downregulation of thyroid stimulating hormone (TSH), with clinical consequences such as weight loss, polyphagia, polydipsia, hyperactivity, aggression, diarrhea, vomiting, and tachycardia (Jones et al. 2019; Peterson and Ward 2007).

In most organisms, THs are transported, bound to the blood serum proteins albumin (ALB), transthyretin (TTR), and thyroxine-binding globulin (TBG) (Richardson 2009). Only TTR and ALB act as TH-binding transport proteins in cats, and TBG has not been found in cat serum (Larsson et al. 1985). ALB is a general transport protein, which occurs in an excessive amount in blood plasma, but ALB has a low specific binding of THs. The transport protein TTR, on the other hand, is highly specific and can transport THs across membranes, such as the blood-brain barrier, across the placenta and into cerebrospinal fluid (Meerts et al. 2002; Schreiber et al. 1995; White and Kelly 2001). Several anthropogenic OHCs have been demonstrated to be able to bind to TTR and competitively inhibit the transport of T4 (Weiss et al. 2015). The majority of the compounds reported to be TTR binders are aromatic and hydroxylated and contain halogens, features they have in common with T4 itself. One compound group that differs structurally but has been shown to bind to TTR are per- and polyfluoroalkyl substances (PFASs) (Weiss et al. 2009).

PFASs exhibit high surface activity due to their hydrophobic and hydrophilic characteristics, and the many carbon-fluor bonds make them extremely stable. This gives them useful properties, e.g., thermal stability, acid resistance, and ability to lower surface tension. Due to this, PFASs can be found in a wide range of industrial and commercial products, such as polymers, surfactants, lubricants in textile coatings, non-stick coatings, stain repellents, food packaging, and firefighting foams (Buck et al. 2011; Wang et al. 2017). The human exposure pathways are mainly via food and drinking water, although PFASs can also diffuse directly from applied household products and associate to dust particles (Vestergren and Cousins 2009). Recent studies have indicated a correlation between concentrations of PFASs in household dust and human serum, particularly for small children (Beesoon et al. 2012; Wu et al. 2015). PFASs have been determined in cat serum from the USA at levels similar to the US population based on the NHANES (National Health and Nutrition Examination Survey) database (Bost et al. 2016; Wang et al. 2018).

The aim of this study was to determine the PFASs used in common consumer products in paired samples of household dust and serum of domestic cats from a central region of Sweden. The samples analyzed are part of a larger project that aimed to identify and evaluate mixtures of thyroid hormone disruptors in our indoor environment (MiSSE—Mixture Assessment of EDCs, Formas 210-2012-131). The PFASs analyzed are perfluoroalkyl carboxylic acids (PFCAs), perfluoroalkyl sulfonic acids (PFSAs), perfluoroalkyl phosphonic acids (PFPAs), perfluoroalkyl phosphinic acids (PFPIAs), polyfluoroalkyl phosphate mono- and diesters (PAP and diPAPs), 6:2 fluorotelomer sulfonic acid (6:2 FTSA), and perfluorooctane sulfonamide (FOSA).

## Materials and methods

### Samples

Seventeen families in Stockholm and Uppsala region (Sweden) participated in the study. Samples were obtained between August 2013 and March 2014. The inclusion requirements of families included having a healthy pet cat and at least one child under 10 years old living at home. The details of participating households and their cats have been reported elsewhere (Norrgran Engdahl et al. 2017). In total, blood was drawn from 29 unsedated pet cats in their home environment. Serum from two pairs of siblings from two homes was pooled due to low sample volume. Thus, 27 cat serum samples (25 individual samples and two pooled samples) from 17 homes are reported. On average, families had two children living at home (1–13 years old) and more than one cat. The majority of cats were domestic (shorthair) cats (57%), and most of the families lived in free-standing houses (53%). The majority of cats (86%) spent more than 50% of their time indoors. The cats’ health was checked by a simple clinical examination before blood was sampled. Their thyroid status was evaluated by measuring levels of total serum T4 and TSH at the laboratory of the university animal hospital at the Swedish University of Agricultural Sciences. All cats were clinically healthy and not on medication.

Blood was taken from the cephalic vein in the right foreleg with a 0.8-mm (21 G) needle into plain evacuated tubes with a clotting activator (BD Vacutainer, BD, Plymouth, UK). Serum was obtained by letting whole blood coagulate at ambient temperature for at least 30 min and then centrifuged for 5 min (3000 G), and the supernatant was collected and stored at −20°C prior to analysis.

Blood lipid content was determined enzymatically for triglycerides and cholesterol. Total lipid was calculated using an average molecular weight for triglycerides and cholesterol of 807 and 571 g/mol, respectively, and assuming free and esterified cholesterol in a ratio of 1:2 (Covaci et al. 2006). Triglycerides and cholesterol were measured at the laboratory of the university animal hospital at the Swedish University of Agricultural Sciences (Norrgran Engdahl et al. 2017).

Still-standing dust was collected using a dust collector (Dustream®, Indoor Biotechnologies Ltd., Wiltshire, UK) containing a disposable filter (mesh size 40 μm) attached to a household vacuum cleaner tube. Rooms sampled were the living room, the adult’s bedroom, the child’s room, and if available, an additional playroom of the participating families. The samples were sieved (1 mm) to isolate the estimated ingested fraction for cats (0.04–1.0 mm). Larger particles such as food crumbs, gravel from outside, or hair were avoided. Due to limited sample amount and other chemical analysis performed within the project, not all of the rooms from the 17 families could be analyzed for PFASs. Dust samples from the following rooms were available: child’s room (*n*=13), adult’s bedroom (*n*=14), living room (*n*=14), and additional play room (*n*=5). In total, 46 dust samples were analyzed, representing all 17 homes.

### Chemical analysis

All PFAS abbreviations are taken from Buck et al. 2011 (Buck et al. 2011). The internal standards (IS) used for quantifying dust and serum levels, with the corresponding native compounds quantified with each isotope in brackets, are ^13^C_4_-PFBA (PFBA), ^13^C_5_-PFPeA (PFPeA), ^13^C_2_-PFHxA (PFHxA), ^13^C_4_-PFHpA (PFHpA), ^13^C_4_-PFOA (PFOA), ^13^C_5_-PFNA (PFNA), ^13^C_2_-PFDA (PFDA), ^13^C_2_-PFUnDA (PFUnDA), ^13^C_2_-PFDoDA (PFDoDA, PFTrDA, PFTeDA), ^18^O_2_-PFHxS (PFBS, PFHxS), ^13^C_4_-PFOS (total PFOS, i.e., branched and linear), ^13^C_2_-6:2 FTSA (6:2 FTSA), ^13^C_4_-6:2 diPAP (6:2 diPAP, 6:6 PFPIA, 6:8 PFPIA), ^13^C_4_-8:2 diPAP (8:2 diPAP, 8:8 PFPIA), ^13^C_2_-6:2 PAP (6:2 PAP), ^13^C_2_-8:2 PAP (8:2 PAP), and Cl-PFHxPA (PFHxPA, PFOPA, PFDA). ^13^C_5_-PFHxA and ^13^C_8_-PFOA were used as recovery injection standards. All analytical standards were purchased from Wellington Laboratories. The calibration curve (concentration range 0.02–5 ng/mL), IS mixture, and injection standards were prepared in methanol (MeOH, J.T Baker). The PFSA, PFCA, 6:2 FTSA and FOSA, and the organophosphorus PFASs (OP-PFASs; PAPs and diPAPs, PFPAs, and PFPIAs) needed different sample pre-treatment and chemical analysis settings, specified below.

### Serum

Fifty microliter cat serum was used for the PFSA, PFCA, 6:2 FTSA, and FOSA analysis. Before sample treatment, the IS mixture (50 μL, concentration 10 ng/mL) and MeOH (150 μL) were added. A larger volume of cat serum (120–520 μL) was used for the OP-PFAS analysis; the IS mixture (50 μL) and MeOH with 1% formic acid (400 μL, Sigma-Aldrich) were added. All samples were vortexed 30 s and sonicated 10 min, followed by centrifugation (13000 rpm) for 10 min. The supernatant was transferred to a vial, and the whole sample was injected into an online system containing a 5 μm C8 solid-phase extraction (SPE) column (4.6 * 10 mm, XTerra MS Waters). The details of the solvent and pump conditions are given in supporting information in Additional file 1 (Table SI-2).

#### Dust

The IS mixture (50 μL, concentration 20 ng/mL) was added to the sieved dust (50 mg). The sample was extracted twice with 2 mL followed by 1 mL MeOH, with 30-s vortex and 15-min sonication in between. The combined extract was centrifuged (3000 rpm) for 10 min, and the supernatant was transferred into a new tube. The extract (3 mL) was split into two equal portions for separate cleanup procedures for analyses of PFSA, PFCA, 6:2 FTSA and FOSA, and OP-PFASs.

The extract for PFSA, PFCA, 6:2 FTSA, and FOSA analyses was diluted in 25 mL water (Milli-Q, obtained from a Milli-Q Reference A+ purification system, Millipore, Bedford, MA, USA), and the pH level was adjusted with ammonium hydroxide (NH_4_OH, Sigma-Aldrich) to pH 10. For liquid/liquid extraction, hexane (4 mL, J.T. Baker) was added to the water, mechanically shaken for 10 min, and subsequently the two phases were separated by centrifugation (3000 rpm). The organic layer was discarded. The pH of the water phase was adjusted to pH 4 by adding hydrochloric acid (Sigma-Aldrich). For cleanup, SPE cartridges (Oasis WAX 150 mg 6cc, Waters) were used. After conditioning the cartridges and loading the extract, the cartridges were washed with 4 mL ammonium acetate (NH_4_Ac, pH 4, Sigma-Aldrich) and 8 mL tetrahydrofuran (Biosolve)/MeOH (3/1, v/v), and the extract was eluted with 4 ml methanol 0.1% NH_4_OH. The eluate was evaporated to dryness and reconstituted in 200 μL 50/50 MeOH/Milli-Q water (v/v).

The extract for OP-PFAS analysis was cleaned up by adding 20 μL acetic acid (and 20 mg ENVI Carb (Supelco) to the extract. The sample was vortexed for 30 s and centrifuged (3000 rpm) for 10 min. The extract was transferred to a new tube, evaporated until dryness, and reconstituted in 200 μL 50/50 MeOH/Milli-Q water (v/v).

The PFAS analysis in dust and serum extracts was performed using liquid chromatography coupled to tandem mass spectrometry (LC-MS/MS). The LC system was an Agilent 1200 Series (Palo Alto, CA, USA) coupled with an Agilent 6410 electro spray interface (ESI) operated in the negative ion mode prior to triple-quadrupole mass spectrometric detection. The details regarding LC and MS settings for cat serum (Table S3 and Table S4) and dust (Table S5 and Table S6) analysis are reported in detail in Additional file 1. The MS was operated in the electrospray negative ionization mode (ESI-) using two multiple reaction monitoring (MRM) transitions for each compound (Table S7 and Table S8). The total PFOS concentration was determined, i.e., the linear and the branched peaks were integrated together. The other branched isomers were not separated.

### QA/QC

In parallel with the samples, solvent blank samples (*n*=10 for dust analysis and *n*=5 for cat blood analysis) were analyzed to determine background contamination. In addition, two serum sampling tubes were extracted and analyzed for possible leaching of PFASs from the material. To control for recovery, reproducibility, and accuracy, reference materials were included in the analysis, consisting of enriched (3 ng/mL) fetal bovine serum (50 μL, *n*=5, PAA laboratories, Pasching Austria) and NIST reference dust SRM2585 (50 mg, *n*=10). The results of the analytical quality assurance (QA) and quality control (QC) of the blood serum and dust sample analyses are reported in detail in Additional file 1 (Table S9-14 and Figure S1).

Average recoveries obtained in bovine serum enriched with PFSA, PFCA, 6:2 FTSA, and FOSA (at levels of 3 ng/mL) varied between 86 and 114% (relative SD <15%, *n*=8) depending on the PFAS (Table S9). For OP-PFASs, recoveries were determined in bovine serum enriched at two levels, 0.16 and 0.74 ng/mL. Three different tests to determine the influence of when to add the IS solution to the samples were performed and are reported in Additional file 1 (Figure S1). Recovery ranged from 50 for PFDPA to >200% for PFHxPA. Labeled IS recovery in the samples ranged between 32 and 259% (Table S11). Due to low recovery (<20%) of IS Cl-PFHxPA for PFPA and PFPIA quantification, these values are not reported.

The method limit of detection (LOD) was set to the lowest calibration concentration with a peak height larger than signal to noise of 3. The limit of quantification (LOQ) was set to 3 times the LOD or the average blank level + 3 times the standard deviation (SD) of the measured PFAS levels in solvent blank samples. The LOQs ranged between 0.2 and 170 pg/mL in blood serum (Table S10) and from 0.01 to 31 ng/g in dust (Table S14) depending on the specific PFAS.

The dust reference material SRM2585 was analyzed in replicates (*n*=8) and compared to PFAS levels reported in literature (Reiner et al. 2015). Accuracy was between 78% and 133% for PFSA and PFCA (Table S12). At the time, there was no literature data on the presence and levels of OP-PFASs in the SRM2585 reference material, and therefore, OP-PFASs were added to the material to evaluate the accuracy (between 3 and 3800 ng/g dust). Recovery of the OP-PFAS fortified samples ranged between 76 and 104% (Table S13). The standard deviation of all PFASs analyzed in the reference material was <20%, indicating a good reproducibility (Table S12 and S13).

### Statistical analysis

The data was checked for potential outliers using Tukey’s outer fence (Foreman 2014), but using 6 × IQR (interquartile range) instead of the original suggested 3 × IQR to get the filter more conservative. Despite this, several extreme values were found outside this range. To achieve a robust test, not influenced by these extremes, Spearman’s coefficient of rank correlation (*r*_S_) was used to correlate between serum PFAS concentrations (molar basis) and serum TH and cholesterol levels. For cases without obvious outliers, ordinary log-linear regression analysis and Pearson correlation (*r*) and the Coefficient of determination *r*^2^, were applied. Corrections for ties were applied according to Zar 1999 (Zar 1999). *p*-values were calculated according to Press et al. (1996). Only positive correlations between serum and dust concentrations were assumed; therefore, one-tailed tests were applied. For the statistical evaluation, values >LOD but <LOQ the LOQ values (for that batch analysis) divided by square root of 2 were used. The non-parametric paired Wilcoxon signed-rank test (Wilcoxon 1945) was used to check for significant differences among rooms within the same homes. Multiple regression analysis (e.g., Kleinbaum et al. 2008) was used to study the potential effects of several PFASs on T4 levels. Principal component analyses (PCA e.g. Varnosa and Filzmoser 2009) were used to disclose potential patterns in relative PFAS concentrations among room types. A significance level of 5% (*α* = 0.05) was applied in the statistical analyses. The statistical analyses were carried out using the software package PIA (Bignert 2013).

## Results

Table 1 summarizes the PFAS concentrations determined in cat serum and household dust. In Additional file 1 (Table S15-25), the detailed information is reported on all individual blood serum and dust samples analyzed.

**Table 1:** PFAS concentrations determined in cat serum (*n*=27) and household dust (*n*=46)

	Cat serum					Household dust				
	>LOD	>LOQ	Mean	Median	Min-max	>LOD	>LOQ	Mean	Median	Min-max
PFAS	(%)		(pg/mL)			(%)		(ng/g dust)		
PFBA	0	0	-	-		11	0	2.0	<LOQ	<LOD-23
PFPeA	0	0	-	-		7	0	0.93	<LOQ	<LOD-23
PFHxA	0	0	-	-		96	52	13	6.4	<LOD-99
PFHpA	96	78	1200	580	<LOD-4300	57	24	5.3	2.2	<LOD-95
PFOA	100	96	1800	1100	<LOQ-15000	100	93	51	9.0	<LOQ-650
PFNA	93	70	360	220	<LOD-1000	93	54	6.2	3.4	<LOD-44
PFDA	100	100	500	430	130-1500	96	52	10	3.2	<LOD-100
PFUnDA	100	96	620	490	<LOQ-1700	54	17	1.9	1.0	<LOD-22
PFDoDA	96	70	140	120	<LOD-290	41	24	6.5	<LOQ	<LOD-110
PFTrDA	0	0	-	-		22	4	0.85	<LOQ	<LOD-9.1
PFTeDA	0	0	-	-		30	17	4.1	<LOQ	<LOD-56
PFBS	0	0	-	-		2	2	0.19	<LOQ	<LOD-8.7
PFHxS	96	93	560	460	<LOD-2100	24	7	0.61	<LOQ	<LOD-8.1
PFHpS	41	22	36	<LOQ	<LOD-170	0	0	-	-	
PFOS	100	100	2300	2300	680-5000	100	93	22	13	<LOQ-220
FOSA	7	4	5.0	<LOQ	<LOD-100	0	0	-	-	
6:2 FTSA	0	0	-	-		65	30	10	1.8	<LOD-220
6:2 PAP	24	16	15	<LOQ	<LOD-130	100	87	67	31	<LOQ-460
8:2 PAP	8	4	4.8	<LOQ	<LOD-75	100	93	47	22	<LOQ-360
6:6 PFPIA	16	16	0.5	<LOQ	<LOD-17	39	26	1.0	<LOQ	<LOD-16
6:8 PFPIA	28	28	2.2	<LOQ	<LOD-5.2	48	33	1.7	<LOQ	<LOD-27
8:8 PFPIA	12	12	0.12	<LOQ	<LOD-1.1	61	35	0.73	0.10	<LOD-11
6:2 diPAP	68	40	11	5.3	<LOD-130	100	100	140	65	11-1300
8:2 diPAP	92	48	13	8.7	<LOD-62	100	100	100	49	7-920
PFHxPA	n.d.		-	-		39	20	3.3	<LOQ	<LOD-31
PFOPA	n.d.		-	-		70	41	47	3.3	<LOD-1800
PFDPA	n.d.		-	-		43	28	8.7	<LOQ	<LOD-70
Σ PFASs			7600	6400	1600-23000			550	280	50-2400

*n.d.* not determined

### PFASs levels in cat serum

All samples (*n*=27) were analyzed for PFCAs, PFSAs, FOSA, and 6:2 FTSA levels, but two samples were lost during analysis of PFPAs, PFPIAs, and PAPs (*n*=25). Due to limited sample volume, no repeated analysis could be performed. PFOS, PFOA, PFDA, and PFUnDA could be detected in all cat blood samples. The highest median concentration was found for PFOS (2300 pg/mL), followed by PFOA (1100 pg/mL). Concentrations of PFPIAs and PAPs were an order of magnitude lower than PFSA/PFCA. The 6:2 and 8:2 diPAPs were detected in 68% (median 5.3 pg/mL) and 92% (median 8.7 pg/mL) of the samples, respectively. The 6:2 and 8:2 PAP concentrations could only be detected in 24% and 8% of the samples, respectively. Three perfluoroalkyl phosphinic acids (6:6-, 6:8-, and 8:8 PFPIA) could be determined in 12–28% of the cat serum samples. Unfortunately, the analytical recovery of the internal standard Cl-PFHxPA was too low (Table S11) during analysis of the cat serum to be able to quantify the PFPA. The levels of sum of all PFASs (ΣPFASs) in the cat serum ranged between 1600 and 23000 pg/mL.

### PFASs levels in household dust

**Fig. 1:**
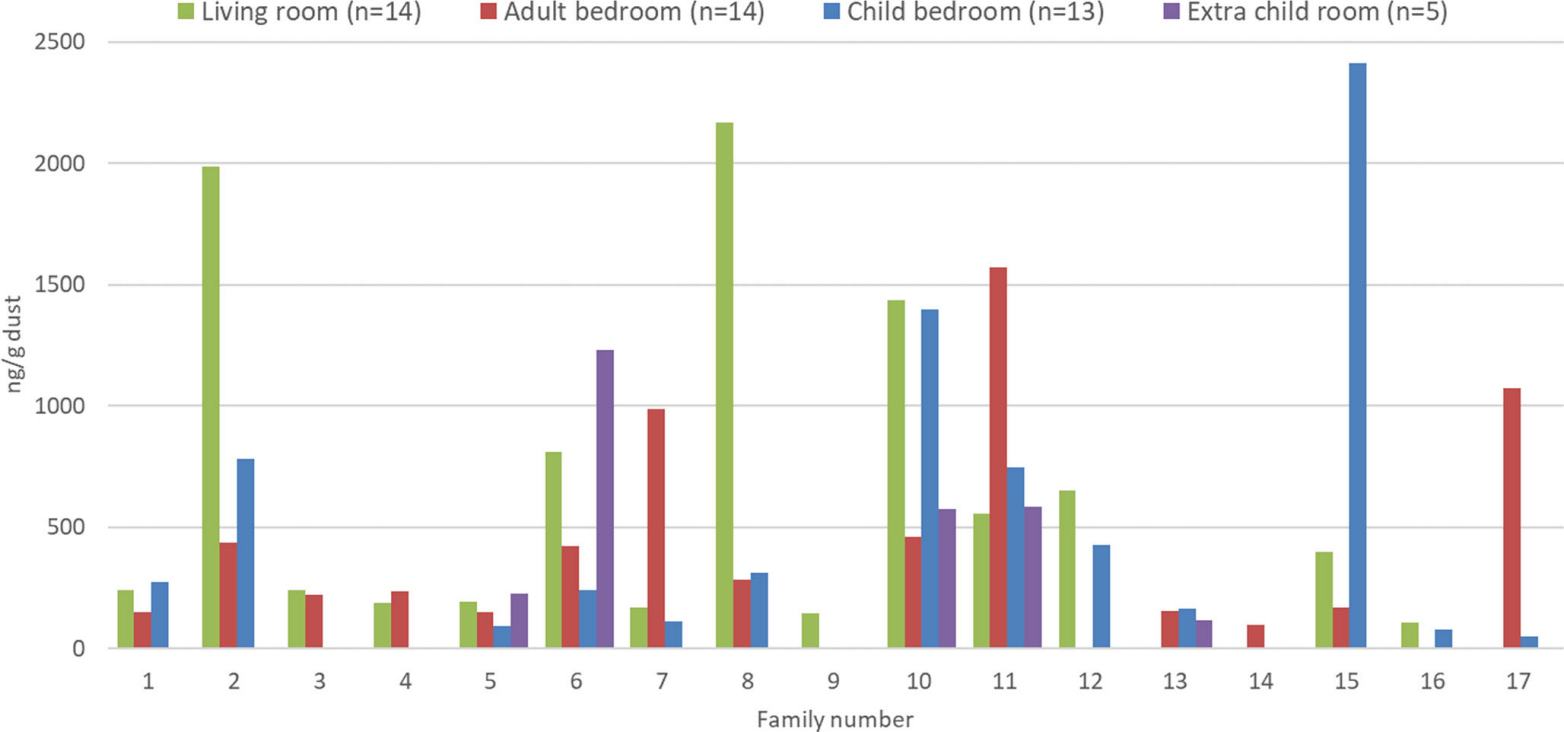
∑PFAS levels (ng/g) in the dust from different rooms sampled at the 17 participating families

PFOA and PFOS were detected in the dust from all rooms and quantified in 93% of the rooms. Median PFOA level was 9 ng/g (max 650 ng/g), and median PFOS level was 13 ng/g (max 220 ng/g). PFNA and PFDA were detected in 100% of the dust samples from the living rooms, but to a lesser extent in the other rooms (Table S18). 6:2 and 8:2 diPAPs were detected and quantified in all rooms from all homes, whereas the 6:2 and 8:2 PAPs were detected in all rooms and quantified in 87% and 93% of the rooms. The median concentrations for diPAPs (65 and 49 ng/g for 6:2 and 8:2 diPAP, respectively) are higher than for PFOA and PFOS, which are dominating the profile for PFCA and PFSA, respectively. PFOPA was detected in 70% of the dust from all rooms but only quantified in 41% of the samples with a median of 3.3 ng/g. There was a larger difference regarding OP-PFAS concentrations between families than for PFCA and PFSA. One family had elevated levels in the living room, with a PFOPA level of 1800 ng/g dust (family 8, Table S18, Fig. 1). The highest ∑PFASs level was found in the child’s bedroom of family 15, due to elevated diPAP levels (Table S23). Family 2 reported elevated levels of both PAPs and diPAPs in the living room (Table S19).

Statistical significance was found when applying the Wilcoxon signed-rank test, which is avoiding variation among homes, hence increasing the possibility to detect potential differences among rooms within the same homes. The test showed that 8:8 PFPIA concentrations were higher in children’s rooms compared to adults’ and also that PFHxA and PFUnDA concentrations were lower (Figure S2). Further, 8:2 PAP concentrations were indicated to be higher in living rooms compared to children’s rooms (Figure S3), as well as 6:2 FTSA concentrations compared to adults’ rooms (Figure S4). Although the concentrations differed between rooms for PFASs, there was no significant difference regarding the PFAS profiles between rooms (Figure S5).

### PFASs levels in household dust vs. cat serum

**Fig. 2:**
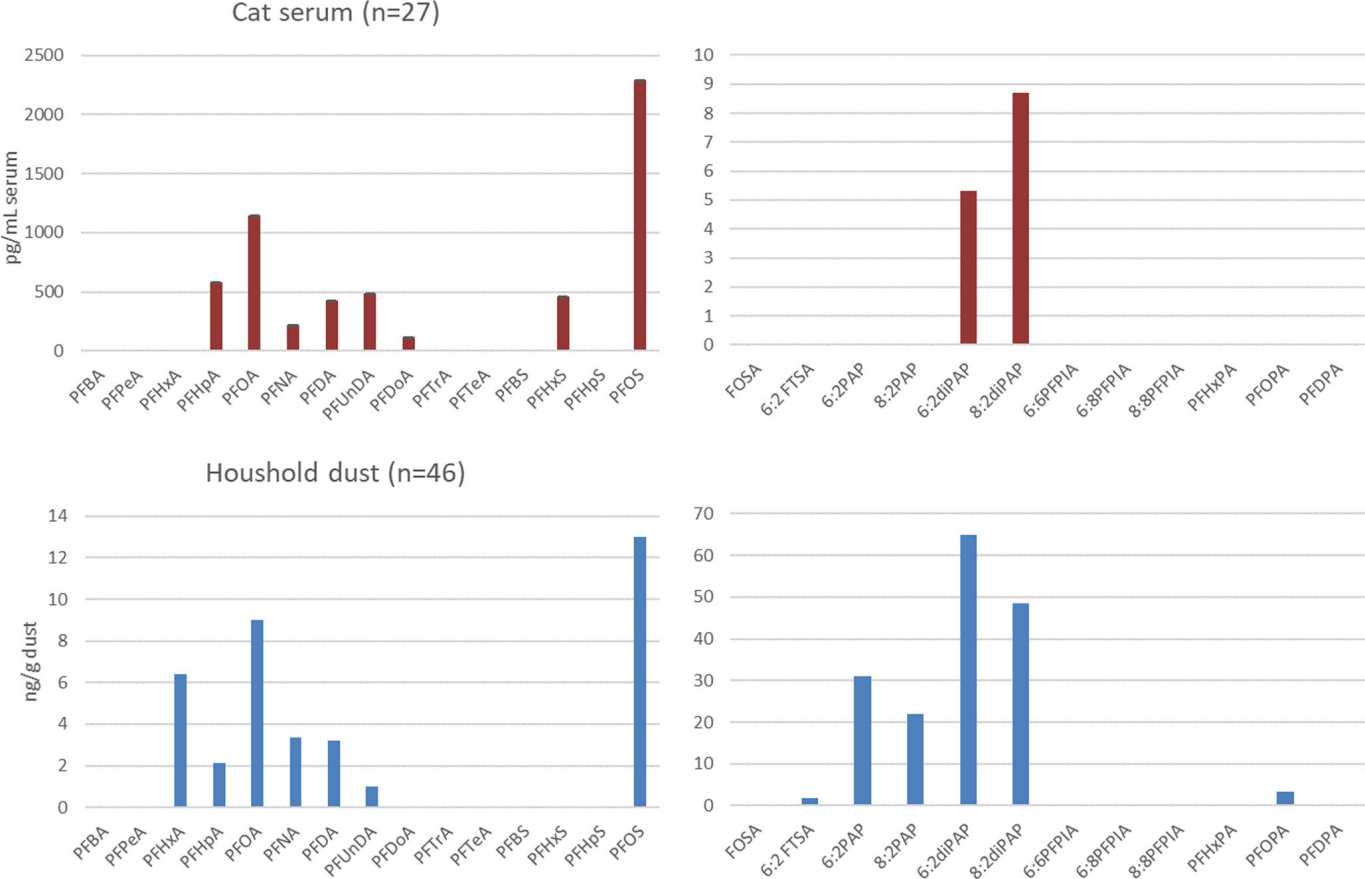
PFAS profile (median) in cat serum (pg/mL) and household dust (ng/g dust)

The levels measured in household dust and in cat blood serum were statistically tested for association between the two matrices, evaluating whether dust is an exposure pathway to cats. In general, the PFAS profile showed resemblances between the two matrices, although the relative concentrations of different PFAS groups differed (Fig. 2). The most significant difference was the dominance of PFOS and PFOA in cat serum and of PAPs and diPAPs in household dust.

Only 8 PFASs (PFHpA, PFOA, PFNA, PFDA, PFUnDA, PFOS, 6:2 diPAP, and 8:2 diPAP) were detected (>LOD) in more than 50% of both cat serum and house dust and were paired for comparison (Figure S6 and S7). As there was no difference between the rooms, the average PFAS dust level was used to compare with the cat(s) living in that household. Serum concentrations were not age-adjusted as no significant correlation could be found with age, although PFOS (*p*=0.068) and PFHxS (*p*=0.090) were close to being significant. All levels were compared using molar-based concentrations. Significant positive correlations were only found for PFOA (*r*_S_=0.32, *p*<0.049) and PFUnDA (*r*_S_=0.55, *p*<0.001), confirming that dust is a relevant PFAS exposure pathway for cats.

### PFASs levels in cat serum *vs.* thyroid hormone levels

**Fig. 3:**
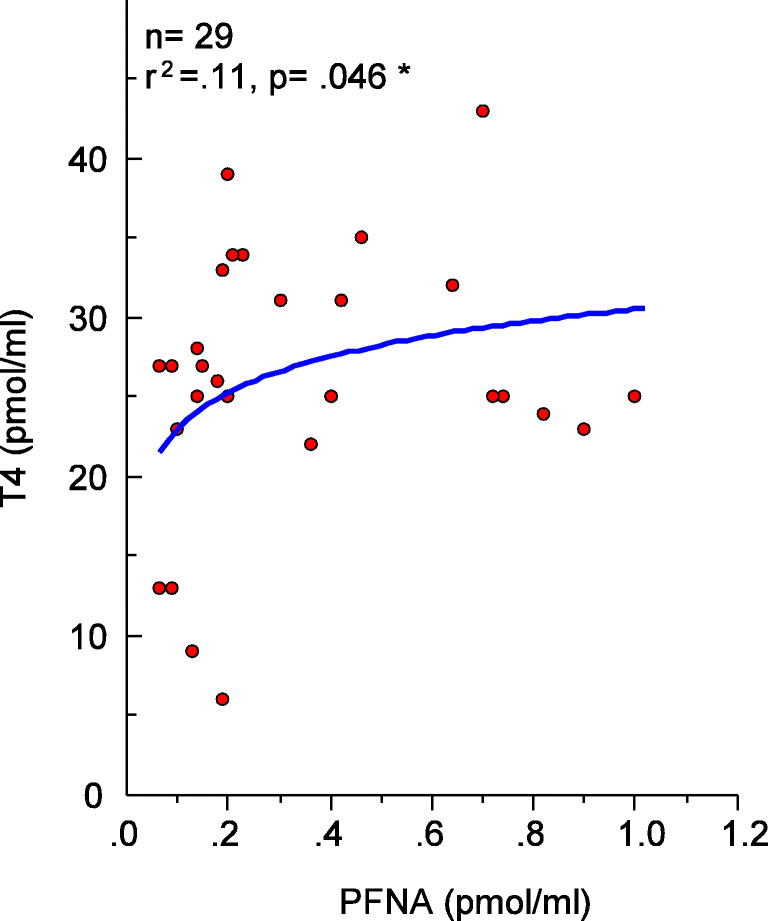
Correlation between serum T4 levels (pmol/mL) and PFNA T4 equivalence (pmol T4 eq/mL) in serum

The cats represented a wide range of breed, age, and size (Norrgran Engdahl et al. 2017). The cats were all healthy, and measured thyroid hormone status (total T4 and TSH) was within a normal range (Table S15). A significant positive increase of serum total T4 levels could be observed with increasing PFNA levels (Fig. 3). The ratio between T4 and TSH levels is sometimes a more sensitive indication to changes in the thyroid hormone system due to feedback regulation, e.g., lowered T4 will increase the response in TSH and vice versa. It has been shown that PFASs bind to the T4 transport protein TTR (Weiss et al. 2009). Here we calculated the total T4 equivalency (T4 eq) by multiplying the T4 relative potency (T4 REP_i_) determined in a radioligand binding assay (Weiss et al. 2009) by the individual PFAS concentrations (*C*_i_) and sum it up for the individual serum samples (T4 eq = Σ[*C*_i_ × T4 REP_*i*_]). Both the serum PFAS and total T4 eq levels were compared to T4, TSH, or the ratio between the two, but no correlations could be seen (Figure S8).

### PFASs levels in cat serum vs. cholesterol levels

**Fig. 4:**
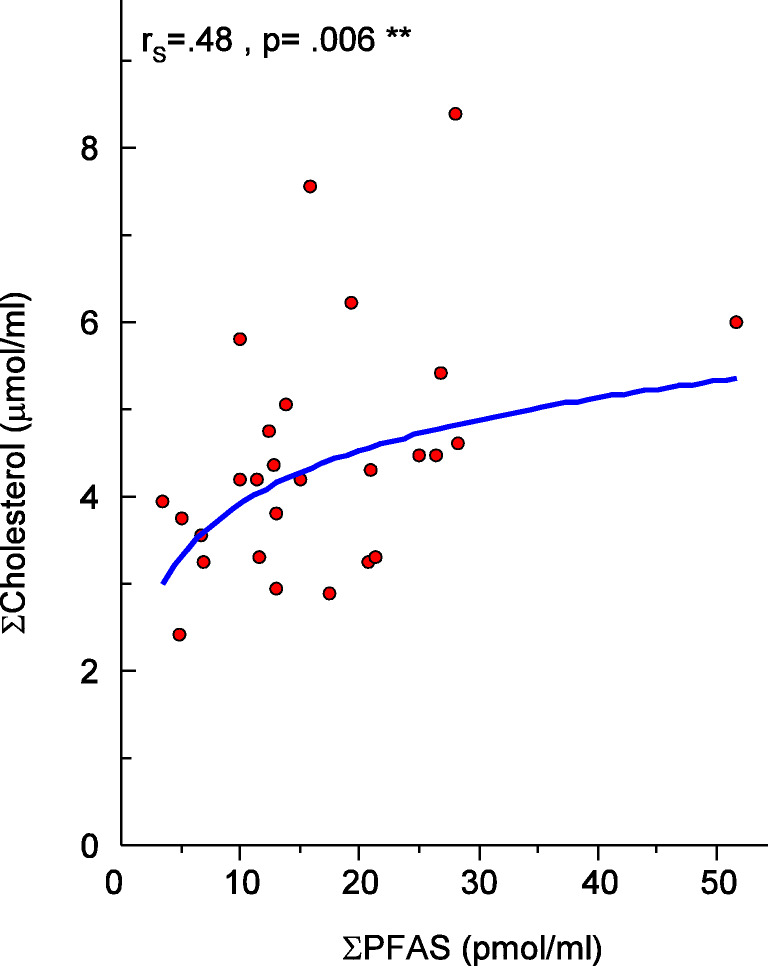
Correlation between serum cholesterol levels (μmol/mL) and PFAS (pmol/mL) in serum

EFSA recently established health-based guidance values for PFOS and PFOA, where one of the critical effects were the increase in serum total cholesterol (EFSA 2018). Mainly PFOS and PFOA have been studied regarding how blood lipid metabolism is affected by PFASs. Here we could not see a statistically significant increase of the cholesterol associated to PFOS and PFOA, although to the total PFASs levels (Fig. 4), as well as with PFHpA, PFUnDA, and PFDoDA (Figure S9). No correlations between cat serum triglycerides and PFASs (individual and sum) were found.

## Discussion

**Fig. 5:**
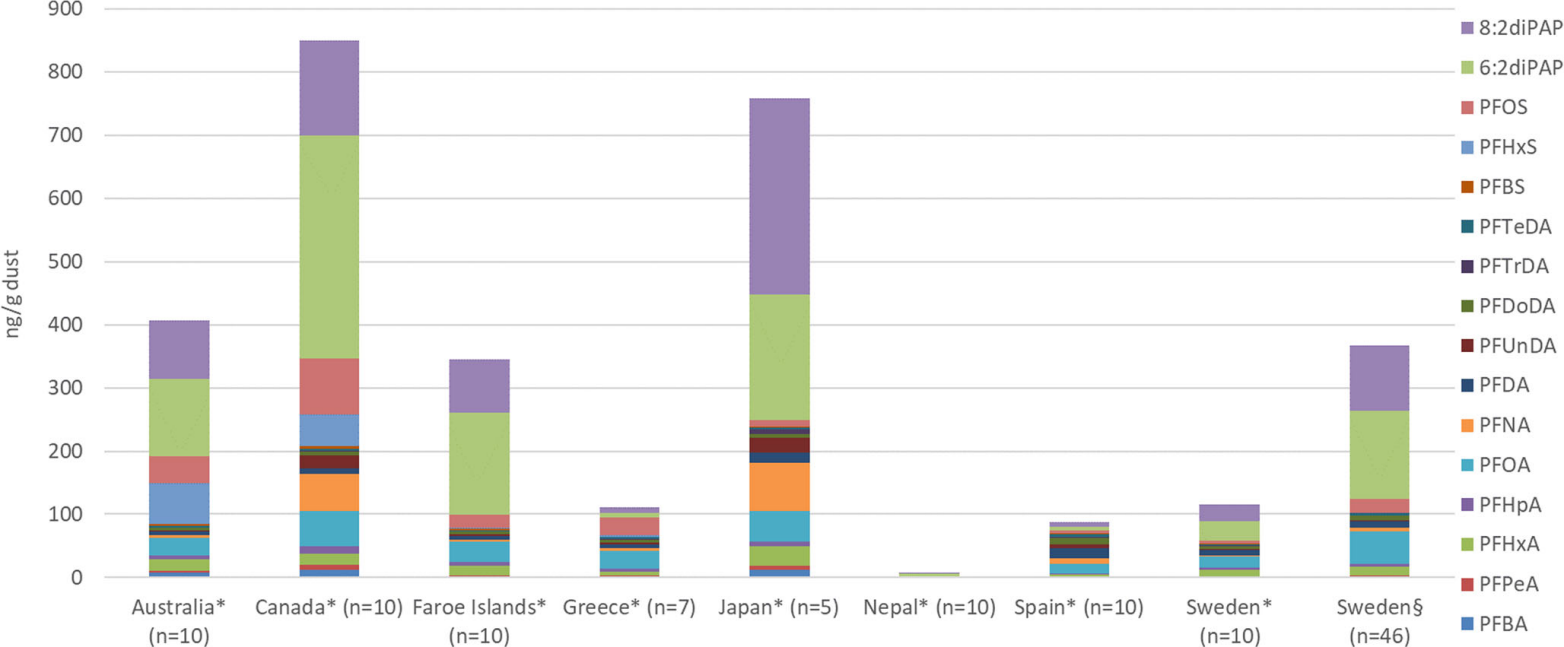
Dust PFAS levels (ng/g dust) compared between this study and one similar study (Eriksson and Kärrman 2015). Selected analytes have been determined in all samples, and the sampling years are 2013–2014. Asterisk shows the average concentration determined in pooled dust from vacuum cleaner consisting of “n” numbers of bags. The analyzed particle size fraction is <150 μm. § shows the arithmetic mean PFAS concentration of the dust sampled in this study (*n*=46) and consist of still-standing dust of particle size 40–1000 μm.

PFAS levels in household dust were within the same range as previously reported. It can be a challenge to compare PFAS levels determined in dust from different studies, as levels are highly influenced by sampling design, e.g., how the dust is collected, the analyzed sample size fraction, region, as well as sampling year. Here we chose to compare our reported PFAS levels with a study where pooled household dust from 8 countries was sampled worldwide in the same sampling year as our study (2013–2014) and analyzed for a wide range of PFASs (Fig. 5) (Eriksson and Kärrman 2015). The particle size fraction was smaller (<150 μm) than in our study (40–1000 μm), which could influence the reported results. An increasing surface-to-area ratio with decreasing particle size can adsorb more analytes (Weiss et al. 2018). Australia, Faroe Islands, and Sweden showed similar PFAS profiles in dust. Except Japan and Canada, the other countries have no PFAS production sites, and the results indicate that consumer products used in the homes are significant sources of PFASs (Wang et al. 2014). Dust from Canada and Australia contained higher dust concentrations of PFOS and PFHxS, chemicals typically used as surfactants in firefighting foams, for example. PFNA was higher in Canada and Japan, a chemical used for the production of polyvinylidene fluoride (Wang et al. 2014). 6:2 and 8:2 diPAP dominated the PFAS profiles in dust from all countries, except Greece, Nepal, and Spain, which were the countries with the lowest total PFAS levels. PAPs were not included in the comparison as they were only semi-quantified in the study. The indicated median levels of PAPs (6:2, 8:2, and 10:2 PAP) in the different countries ranged from 3.7 to 1 023 ng/g, and the levels were dominating the PFAS profile together with diPAPs (∑15 diPAPs, 3.6–692 ng/g). As diPAPs dominates the technical mixture used in products, it is likely that PAPs are products of degradation of diPAPs. (Lee et al. 2010).

**Fig. 6:**
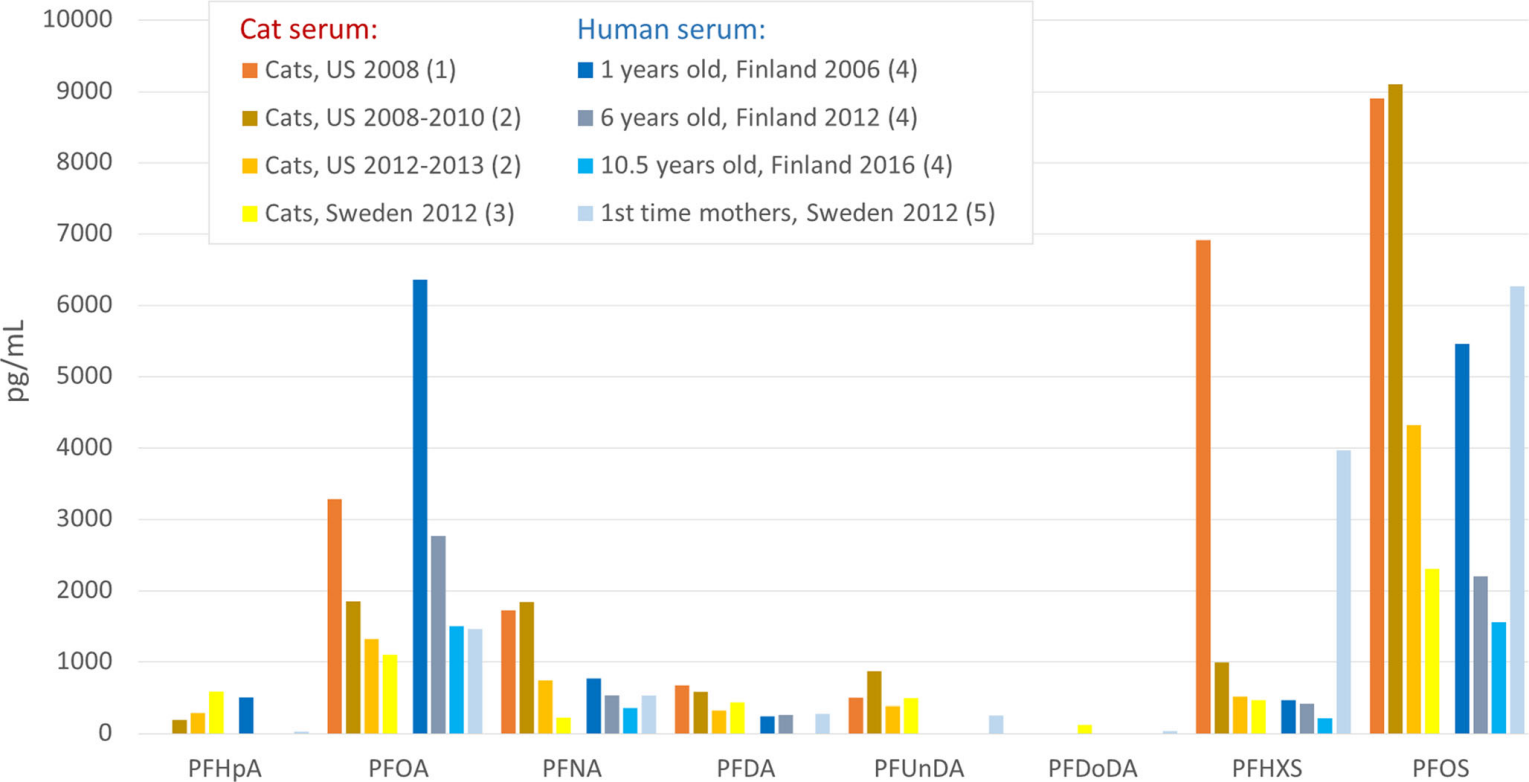
PFAS concentrations determined in cat serum and human serum taken from literature. (1) Geometric mean (Bost et al. 2016). (2) Geometric mean (Wang et al. 2018). (3) This study, median. (4) Average of median for male and female children (Koponen et al. 2018). (5) Pooled blood serum samples (pg/g serum) (Gebbink et al. 2015)

Regarding PFCAs and PFSAs in cat serum, the levels are comparable to other studies reporting on PFASs in cats and humans (Fig. 6). There are only two other studies reporting on PFASs in cats, on cats situated in the USA (Bost et al. 2016; Wang et al. 2018). The levels were higher than in the Swedish cats and seem to be decreasing over time, although the number of samples was too small to draw any conclusions. PFASs were determined in children from Finland at different ages while growing up (Koponen et al. 2018). Levels were decreasing with age, which was explained by the rapid body weight gain whereas the total body load was constant. At the age of 10, the average serum level of PFOA was similar to the median cat serum levels in Sweden, and at the age of 6 for PFOS. Serum has been analyzed from 1st-time mothers coming from the same region as the cats in this study. PFAS levels were similar except for PFHxS and PFOS, which were equally high as in cats in the USA in 2008. This was due to drinking water contamination from a firefighting training site (Ahrens et al. 2015). The cats from this study coming from that specific region did not have elevated PFHxS and PFOS levels, probably due to the fact that cats drink very little water. Their main water supply comes via their food.

Eight PFASs could be paired in the dust and cat serum (Figure S6). Two PFASs, PFOA, and PFUnDA were significantly correlated, demonstrating that dust was a relevant exposure pathway. It is reasonable to speculate that other exposure routes, such as drinking water and cat food, are contributing to cat serum levels, especially for the levels of PFOS and PFHxS commonly found in fish and drinking water (Ahrens et al. 2015). Therefore, the correlations found for PFOA and PFUnDA indicate that dust is an especially relevant exposure pathway for these compounds and that, generally, chemicals associated to dust are ingested.

The dominance of PAPs and diPAPs in dust was not reflected in the cat serum. Only low levels of PAPs and diPAPs were determined in a limited number of serum samples. PFASs, including diPAPs, have been determined in human serum samples from donors in the USA in 2009 (Lee and Mabury 2011). The levels of PFOS and PFOA were comparable to the reported cat serum samples here, but diPAP levels were about 10 times higher and were found in 80% of the samples. This indicates that PAPs and PFSA/PFCA have different sources. PAPs were not determined in the US blood donors. The biotransformation of diPAPs has been studied in Carp (Chen et al. 2019). Several metabolites were detected but not the corresponding PAPs. It has been suggested that PAPs in a typical mammalian digestive tract would hydrolyze in approximately 100 s (Jackson and Mabury 2012). In an occupationally exposed population in China, average 6:2 and 8:2 diPAP levels of 0.19 and 0.17 ng/mL and 6:2 PAP level of 0.11 ng/mL have been reported. PFOA and PFOS levels were considerably higher than in the general population (average of 325 and 1064 ng/mL, respectively), and it is reasonable to believe that the workers were exposed to PAPs and diPAPs in their occupation as well (Gao et al. 2018). Serum, plasma, and whole blood were collected from men and women (*n*=61) living in the Oslo area, Norway, to determine the distribution between the matrices of these surface-active compounds (Poothong et al. 2017). It was observed that the quantification frequency was much higher in plasma than in serum, which is the matrix commonly analyzed from humans. Whole blood had the lowest quantification frequency for PAPs, clearly showing the complexity to extract these compounds from blood proteins.

It is worth noting that the cats in this study were all healthy, and therefore, any statistical association to altered thyroid hormone levels is challenging to find in a study with only a limited number of cats. Despite that, a positive significant association between the serum total T4 and PFNA levels was observed (Fig. 3). In children 1–17 years of age living in the vicinity of a chemical plant, this association was also observed (Lopez-Espinosa et al. 2012). Serum PFOA concentrations were also associated with thyroid disease in those children. In the two studies with cats from the USA, where different endocrine diseases were studied, a significant association between hyperthyroidism and PFOA levels was reported, as well as a weak association with ΣPFAS levels (Bost et al. 2016; Wang et al. 2018). No significant correlation could be determined between PFAS levels and the amount of time the cat spent indoors, as was observed in another study (Bost et al. 2016).

A recent study evaluated dust-related thyroid hormone disrupting compounds in mixtures corresponding to household dust, infant blood serum, and adult blood serum (Hamers et al. 2020). In total, 25 chemicals were measured for their binding potency to the transport protein TTR in a competitive in vitro assay (FITC-T4). An effect level corresponding to the inhibition of the T4-TTR binding of 20% was used in the study, mainly as robust data could be achieved at that level, but also as even small changes in available TH levels can cause severe effects on brain development. Moreover, animal studies with OHCs have reported an association between decreased maternal levels of circulating T4 and neurodevelopmental effects on cognitive function, motor activity, and behavior in offspring (EFSA 2005; EFSA 2011a; EFSA 2011b; EFSA 2018). By applying the toxic unit (TU) summation, which assumes the principle of concentration addition for the investigated 25 chemicals, it was predicted that from 1.3 up to 6.2% of T4-TTR binding in human blood could be inhibited by the chemical mixture tested (Hamers et al. 2020). The calculations took into consideration serum TBG and ALB levels as relevant TH binders in humans, where a 12/71/17% distribution of T4 over TTR/TBG/ALB was calculated. As TBG has not been found in cat serum, it is believed that TTR has a more important role in the transport of TH in cats than in other mammals (Larsson et al. 1985). This study is part of a project abbreviated MiSSE (Mixture Assessment of EDCs), and the same cat serum samples have been measured for a range of OHCs, summarized in Table 2. The TUs are calculated by dividing the measured concentration (median or max) by the effect concentration (IC_20_). If the sum of individual TUs equals 1, the concentration is expected to produce the effect (20% inhibition of T4-TTR binding).

**Table 2:** Calculated toxic units (TUs) based on IC_20_ values in the FITC-T4 in vitro assay (Hamers et al. 2020) and concentrations (nM) determined in this study and a previous study (Norrgran Engdahl et al. 2017)

Substance	IC_20_^a^ (nM)	*C*_median_ (nM)	*C*_max_ (nM)	TU_median_	TU_max_
PFHxS	136	1.2	5.3	0.01	0.04
PFOS	82	4.6	10	0.06	0.12
PFHpA	299	1.6	12	0.01	0.04
PFOA	491	2.8	35	0.01	0.07
PFNA	361	0.47	2.2	0.001	0.01
PFDA	1710	0.84	2.9	0.0005	0.002
PFUnDA	1010	0.87	3.0	0.0009	0.003
Pentachlorophenol ^b^	17	0.41	4.2	0.02	0.24
CB153^b^	77	0.17	1.5	0.002	0.02
2,4,6-TBP^b^	8.9	0.44	1.1	0.05	0.13
6OH-BDE47^b^	31	0.5	3.4	0.02	0.11
BDE47^b^	210	0.04	0.26	0.0002	0.001
BDE99^b^	73	0.06	0.61	0.0008	0.008
∑TUs				0.17	0.79

^a^Hamers et al. (2020)

^b^Norrgran Engdahl et al. (2017)

The ∑TUs for TU_median_ in the cat serum was 0.17, which is a margin of 5 from equal to 1, whereas the ∑TUs for TU_max_ was 0.79. Just as in human blood (Hamers et al. 2020), PFOS was one of the major contributors to the ∑TUs in cat serum. The PFASs in cat serum constituted almost 50% of the ∑TUs for the median levels, and 36% for the max values, despite the rather low binding potency of PFASs (Weiss et al. 2009). It is difficult to extrapolate the in vitro assay results to real-life conditions and to estimate the relevance of this T4 displacement from TTR in cats remains to be clarified.

This exercise only contains 13 compounds determined in the cat’s serum of this project. In the literature, more than 60 OHCs have been reported in cat serum, of which many have been tested for their binding potential to TTR (Weiss and Jones 2020). It is therefore likely to believe that the TU summation here is an underestimation of the real situation for household cats. Only considering the PFASs on the market, PFAS composition is changing in products as regulations change and new PFAS substitutes are entering the market. Today there are believed to be more than 3000 different PFASs circulating the global market, and more than 4500 PFASs are registered with CAS numbers (KemI 2015; OECD 2018; Wang et al. 2017). What yet needs to be evaluated is whether the new PFASs are bioavailable and could pose a threat to health.

Several epidemiological studies have reported associations between PFASs (mainly PFOS and PFOA) and human health outcomes linked to blood lipids (Eriksen et al. 2013; Fletcher et al. 2013; Li et al. 2020; Liu et al. 2020). The underlying mechanisms are discussed, but it is agreed on that interferences with the lipid metabolism may potentially lead to an elevated risk of developing cardiovascular diseases, which is one of the 6 most common diagnostic categories for mortalities in cats (Egenvall et al. 2009). It is out of the scope for this study to speculate why some PFASs are correlating with the serum cholesterol and some are not. It is important to acknowledge that the sample size in this study is limited and hence the results should be understood as indicative and further research is needed before prompt conclusions can be drawn.

## Conclusion

This study confirmed that dust is a relevant exposure pathway to PFASs for cats. Levels are similar in humans and pet cats, and it is plausible that the exposure to certain PFASs can affect the thyroid hormone system, although that needs to be confirmed with further research. The critical health effect is recognized by EFSA; increased total cholesterol in blood could also be observed in cats, although for total PFAS, PFHpA, PFUnDA, and PFDoDA, and not for PFOS and PFOA. The high levels of PAPs and diPAPs in dust samples were not reflected in the cats’ serum, indicating a lower bioavailability, fast excretion, or metabolism to PFCAs. With respect to PFASs, the potential of using cats as a model organism for human exposure has been demonstrated. By combining exposure assessment with health parameters in studying pet animals, we can improve our understanding of the health impacts that exposure to indoor-related chemicals have on us and our pets.

## Supplementary Information

ESM 1 (DOCX 418 kb)

## Data Availability

All data generated or analyzed during this study are included in this published article and the supporting information in Additional file 1.

## Abbreviations

OHC: Organohalogen compound
EDC: Endocrine disrupting compound
TH: Thyroid hormone
T4: Thyroxine
TSH: Thyroid stimulating hormone
TTR: Transthyretin
PFAS: Per- and polyfluoroalkyl substance
PFCA: Perfluoroalkyl carboxylic acid
PFSA: Perfluoroalkyl sulfonic acid
OP-PFAS: Organophosphorus PFAS
PFPA: Perfluoroalkyl phosphonic acid
PFPIA: Perfluoroalkyl phosphinic acid
PAP and diPAP: Polyfluoroalkyl phosphate mono- and diesters
FTSA: Fluorotelomer sulfonic acid
FOSA: Perfluorooctane sulfonamide
LOD: Limit of detection
LOQ: Limit of quantification
SPE: Solid-phase extraction
TU: Toxic unit
