# Supplementary material for: Per- and polyfluoroalkyl substances (PFASs) in Swedish household dust and exposure of pet cats
Source: Environ Sci Pollut Res Int. 2021 Mar 20;28(29):39001–13. doi: 10.1007/s11356-021-13343-5 (PMC8310504; doi:10.1007/s11356-021-13343-5)
Supplement: Supplementary file 1 — (DOCX 418 kb) [file 11356_2021_13343_MOESM1_ESM.docx]

Per- and polyfluoroalkyl substances (PFASs) in Swedish household dust and exposure of pet cats

Jana M. Weiss^1*^, Bernt Jones^2^, Jacco Koekkoek^3^, Anders Bignert^1^, Marja H. Lamoree^3^

^1^ Department of Environmental Science, Stockholm University, Svante Arrheniusväg 12, 10691 Stockholm, Sweden.

^2^ Department of Clinical Sciences, Swedish University of Agricultural Sciences, Box 7054, 75007 Uppsala, Sweden.

^3^ Vrije Universiteit, Department Environment & Health, De Boelelaan 1087, 1081HV Amsterdam, The Netherlands.

* Corresponding author

Table of Contents

[Analytical settings 5](#_Toc63858936)

[Table S 1. Online enrichment on SPE column (C8 5 µm (4.6 * 10 mm), Xterra MS Waters) PFSA, PFCA, 6:2 FTSA and FOSA analysis in cat serum. 5](#_Toc63858937)

[Table S 2. Online enrichment on SPE column (C8 5 µm (4.6 * 10 mm), Xterra MS Waters) for OP-PFAS analysis in cat serum. 5](#_Toc63858938)

[Table S 3. LC-MS settings for PFSA, PFCA, 6:2 FTSA and FOSA analysis in cat serum (analytical column 18 EVO 2.6 µm, 100 * 2.1 mm, Kinetex Phenomenex). 6](#_Toc63858939)

[Table S 4. LC-MS settings for OP-PFAS analysis in cat serum (analytical column C18 EVO 2.6 µm, 100 * 2.1 mm, Kinetex). 6](#_Toc63858940)

[Table S 5. LC-MS settings for PFSA, PFCA, 6:2 FTSA and FOSA analysis in dust (analytical column FluoroSEP-RP Octyl, 150 x 2.1mm, 5µm, ES Industries). 7](#_Toc63858941)

[Table S 6. LC-MS settings for OP-PFAS analysis in dust (analytical column C18 EVO 2.6 µm, 100 * 2.1 mm, Kinetex Phenomenex). 7](#_Toc63858942)

[Table S 7. MS settings (dynamic MRM) of the PFSA, PFCA, 6:2 FTSA and FOSA analysis. 8](#_Toc63858943)

[Table S 8. MS settings (dynamic MRM) of the OP-PFAS analysis. 9](#_Toc63858944)

[Quality Assurance/Quality Control 10](#_Toc63858945)

[Table S 9. The accuracy (recovery of native, %) and relative standard deviation of the PFSA, PFCA, and FOSA analysis of enriched (3 ng/mL) bovine serum (n=8) analyzed together with the cat serum samples. 10](#_Toc63858946)

[Figure S 1. Average recovery (%) of fortified native OP-PFASs in bovine serum samples in three tests and at two levels (duplicates per test). 11](#_Toc63858947)

[Table S 10. Average (pg/mL) method limit of quantification (LOQ) of PFASs in blood serum samples, set to 3xLOD or based on average levels + 3xSD of the measured PFAS levels in solvent blank samples. 12](#_Toc63858948)

[Table S 11. Recovery (%) of the PFAS internal standards in cat serum samples (n=27). 12](#_Toc63858949)

[Table S 12. Average PFSA, PFCA, and 6:2 FTSA concentration (ng/g dust) and the reproducibility (relative standard deviation, RSD %) in quality control dust sample dust (SRM2585, n=8) analyzed simultaneously with the dust samples. The levels are compared (accuracy, %) to reported reference values by Reiner et al. (2011). 13](#_Toc63858950)

[Table S 13. Average levels (ng/g dust) and the reproducibility (relative standard deviation, RSD) of OP-PFAS in quality control (SRM2585) dust samples (n=8) analyzed in parallel with target dust samples. The SRM2585 dust was fortified with a realistic OP-PFAS profile and the accuracy (recovery %) and reproducibility (RSD) of the fortified dust samples (SRM2585, n=8) is reported. 13](#_Toc63858951)

[Table S 14. The average (ng/g dust) LOQ based on 3xLOD or average +3xSD of the measured PFAS levels in solvent blank samples (n=10) analyzed in parallel with the samples. 14](#_Toc63858952)

[Results in details 15](#_Toc63858953)

[Table S 15. Detailed cat serum sample information on cats weight, age, and gender, as well as total thyroxine (tT4), thyroid stimulating hormone (TSH) and ΣPFAS levels (pg/mL). 15](#_Toc63858954)

[Table S 16. Detailed PFCA and PFSA levels (pg/mL) determined in cat serum samples. 16](#_Toc63858955)

[Table S 17. Detailed FOSA and organophosphorus PFAS levels (pg/mL) determined in cat serum samples. 17](#_Toc63858956)

[Table S 18. PFCA and PFCA concentrations (ng/g dust) determined in dust samples from the living rooms. 18](#_Toc63858957)

[Table S 19. 6:2 FTSA and organophosphorus PFAS concentrations and ΣPFASs (ng/g dust) determined in dust samples from the living rooms. 19](#_Toc63858958)

[Table S 20. PFCA and PFCA concentrations (ng/g dust) determined in dust samples from the adult bedrooms. 20](#_Toc63858959)

[Table S 21. 6:2 FTSA and organophosphorus PFASs concentrations (ng/g dust) determined in dust samples from the adult bedrooms. 21](#_Toc63858960)

[Table S 22. PFCA and PFCA concentrations (ng/g dust) determined in dust samples from the child rooms. 22](#_Toc63858961)

[Table S 23. FTSA and organophosphorus PFASs concentrations (ng/g dust) determined in dust samples from the child rooms. 23](#_Toc63858962)

[Table S 24. PFCA and PFCA concentrations (ng/g dust) determined in dust samples from the extra rooms. 24](#_Toc63858963)

[Table S 25. FTSA and organophosphorus PFAS concentrations (ng/g dust) determined in dust samples from the extra rooms. 25](#_Toc63858964)

[Figure S 2. Wilcoxon Signed Rank test (paired). Ordered after level of significance, significant differences to the left of the vertical line (p<0.05). Mean values above 1 implies higher values in children's room. 26](#_Toc63858965)

[Figure S 3. Wilcoxon Signed Rank test (paired). Ordered after level of significance, significant differences to the left of the vertical line (p<0.05). Mean values above 1 implies higher values in children's room. 26](#_Toc63858966)

[Figure S 4. Wilcoxon Signed Rank test (paired). Ordered after level of significance, significant differences to the left of the vertical line (p<0.05). Mean values above 1 implies higher values in adult’s bedroom. 27](#_Toc63858967)

[Figure S 5. Principal component analysis (PCA) on the PFAS profile in dust among various rooms. The larger dots represent a center point and the ellipse a confidence interval in which 95% of the dots are expected to fall within. As the centers of gravity overlap, there is no significant difference among type of rooms. 27](#_Toc63858968)

[Figure S 6. Serum (pmol/mL) vs dust (pmol/g) concentrations of perfluoroalkyl carboxylic (PFHpA, PFOA, PFNA, PFDA, PFUnDA) and perfluorooctane sulfonic acid (PFOS) presented on a logarithmic scale. Spearman's rank correlation (one-tailed tests) is showed (r_s_). 28](#_Toc63858969)

[Figure S 7. Serum (pmol/mL) vs dust (pmol/g) concentrations of 6:2 and 8:2 diPAPs. Spearman's rank correlation (one-tailed tests) is showed (r_s_). 29](#_Toc63858970)

[Figure S 8. Logged concentrations of serum-T4 (nmol/L), TSH (µg/L) and the ratio T4/TSH vs PFAS total T4 equivalents (pmol T4 eq/mL). No significant correlations were found, using Spearman's rank correlation (r_s_), two-tailed tests. 29](#_Toc63858971)

[Figure S 9. Significant correlations found for unlogged PFHpA and logged PFUnDA and PFDoDA concentrations vs. serum cholesterol levels, using Spearman's rank correlation (r_s_), one-sided tests. 30](#_Toc63858972)

###

# Analytical settings

## Table S 1. Online enrichment on SPE column (C8 5 µm (4.6 * 10 mm), Xterra MS Waters) PFSA, PFCA, 6:2 FTSA and FOSA analysis in cat serum.

| **Time (minutes)** | **Step** | **Solvent** |
| --- | --- | --- |
| 0 – 1 | Injection | 0.1M Formic acid, 0.5 ml |
| 1 – 5 | Enrichment and washing | 0.1M Formic acid, 2 ml |
| 5 – 12 | Elution to analytical column | 25 mM NH4Ac pH4 and methanol |
| 12 – 16 | Conditioning | Methanol, 2 ml |
| 16 – 20 | Conditioning | Isopropanol, 2 ml |
| 20 – 25 | Conditioning | 0.1M Formic acid, 2.5 ml |

## Table S 2. Online enrichment on SPE column (C8 5 µm (4.6 * 10 mm), Xterra MS Waters) for OP-PFAS analysis in cat serum.

| **Time (minutes)** | **Step** | **Solvent** |
| --- | --- | --- |
| 0 – 1 | Injection | 0.2% Formic acid, 1 ml |
| 1 – 6 | Enrichment and washing | 0.2% Formic acid, 4 ml |
| 6 – 14 | Elution to analytical column | 0.05% NH4OH and methanol |
| 14 – 16 | Washing | Acetone, 2 ml |
| 16 – 19 | Conditioning | Isopropanol, 2 ml |
| 19 – 21 | Conditioning | Methanol, 2 ml |
| 21 – 25 | Conditioning | Acetone, 4 ml |
| 25 – 30 | Conditioning | 0.2% Formic acid, 5 ml |

## Table S 3. LC-MS settings for PFSA, PFCA, 6:2 FTSA and FOSA analysis in cat serum (analytical column 18 EVO 2.6 µm, 100 * 2.1 mm, Kinetex Phenomenex).

| **Time (minutes)** | **Flow (mL/min)** | **25 mM NH4Ac pH4 (%)** | **Methanol (%)** |
| --- | --- | --- | --- |
| 0 | 0.2 | 75 | 25 |
| 5 | 0.2 | 75 | 25 |
| 6 | 0.2 | 50 | 50 |
| 12 | 0.2 | 0 | 100 |
| 19 | 0.2 | 0 | 100 |
| 19.2 | 0.2 | 75 | 25 |
| 25 | 0.2 | 75 | 25 |

**MS settings:**

Ionization: ESI, negative mode

Gas temperature: 325ºC

Gas flow: 6 L/min

Nebulizer: 25 psi

Capillary: 1000 V

## Table S 4. LC-MS settings for OP-PFAS analysis in cat serum (analytical column C18 EVO 2.6 µm, 100 * 2.1 mm, Kinetex).

| **Time (minutes)** | **Flow (mL/min)** | **0.1 % NH_4_OH (%)** | **Methanol (%)** |
| --- | --- | --- | --- |
| 0 | 0.3 | 90 | 10 |
| 6 | 0.3 | 70 | 30 |
| 7 | 0.3 | 55 | 45 |
| 10 | 0.3 | 10 | 90 |
| 15 | 0.3 | 10 | 90 |
| 15.2 | 0.3 | 0 | 100 |
| 30 | 0.3 | 0 | 100 |

**MS settings:**

Ionization: ESI, negative mode

Gas temperature: 350ºC

Gas flow: 9 L/min

Nebulizer: 35 psi

Capillary: 4000 V

## Table S 5. LC-MS settings for PFSA, PFCA, 6:2 FTSA and FOSA analysis in dust (analytical column FluoroSEP-RP Octyl, 150 x 2.1mm, 5µm, ES Industries).

| **Time (minutes)** | **Flow (ml/min)** | **5 mM NH4HCOO (%)** | **Methanol (%)** |
| --- | --- | --- | --- |
| 0 | 0.3 | 65 | 35 |
| 1 | 0.3 | 65 | 35 |
| 16 | 0.3 | 5 | 95 |
| 21 | 0.3 | 5 | 95 |
| 21.2 | 0.4 | 5 | 95 |
| 29 | 0.4 | 5 | 95 |
| 29.2 | 0.4 | 65 | 35 |

**MS settings:**

Ionization: ESI, negative mode

Gas temperature: 325ºC

Gas flow: 6 L/min

Nebulizer: 25 psi

Capillary: 1000 V

## Table S 6. LC-MS settings for OP-PFAS analysis in dust (analytical column C18 EVO 2.6 µm, 100 * 2.1 mm, Kinetex Phenomenex).

| **Time (minutes)** | **Flow (mL/min)** | **0.1 % NH_4_OH (%)** | **Methanol (%)** |
| --- | --- | --- | --- |
| 0 | 0.3 | 98 | 2 |
| 2 | 0.3 | 98 | 2 |
| 18 | 0.3 | 10 | 90 |
| 18.4 | 0.3 | 10 | 90 |
| 18.5 | 0.3 | 0 | 100 |
| 26 | 0.3 | 0 | 100 |
| 26.3 | 0.3 | 98 | 2 |

**MS settings:**

Ionisation: ESI, negative mode

Gas temperature: 350ºC

Gas flow: 9 L/min

Nebulizer: 35 psi

Capillary: 4000 V

## Table S 7. MS settings (dynamic MRM) of the PFSA, PFCA, 6:2 FTSA and FOSA analysis.

| **Compound** | **Precursor Ion** | **Product Ion** | **Fragmentor** | **Collision E (V)** | **Purpose** | **IS** |
| --- | --- | --- | --- | --- | --- | --- |
| PFBA | 213.0 | 169.0 | 60 | 3 | Quant | ^13^C_4_ PFBA |
| ^13^C_4_ PFBA | 217.0 | 172.0 | 60 | 3 | IS |  |
| PFPeA | 263.0 | 219.0 | 60 | 3 | Quant | ^13^C_5_ PFPeA |
| ^13^C_5_ PFPeA | 268.0 | 223.0 | 60 | 3 | IS |  |
| PFHxA | 313.0 | 269.0 | 80 | 3 | Quant | ^13^C_2_ PFHxA |
| PFHxA | 313.0 | 119.0 | 80 | 3 | Qualifier |  |
| ^13^C_2_ PFHxA | 315.0 | 270.0 | 80 | 3 | IS |  |
| PFHpA | 363.0 | 319.0 | 70 | 4 | Quant | ^13^C_4_ PFHpA |
| PFHpA | 363.0 | 169.0 | 70 | 12 | Qual |  |
| ^13^C_4_ PFHpA | 367.0 | 321.9 | 71 | 4 | IS |  |
| PFOA | 413.0 | 369.0 | 72 | 4 | Quant | ^13^C_4_ PFOA |
| PFOA | 413.0 | 169.0 | 72 | 12 | Qual |  |
| ^13^C_4_ PFOA | 417.0 | 371.9 | 72 | 4 | IS |  |
| PFNA | 463.0 | 419.0 | 71 | 4 | Quant | ^13^C_5_ PFNA |
| PFNA | 463.0 | 219.0 | 71 | 12 | Qual |  |
| ^13^C_5_ PFNA | 468.0 | 422.9 | 72 | 4 | IS |  |
| PFDA | 513.0 | 469.0 | 69 | 4 | Quant | ^13^C_2_ PFDA |
| PFDA | 513.0 | 219.0 | 69 | 12 | Qual |  |
| ^13^C_2_ PFDA | 515.1 | 469.9 | 70 | 4 | IS |  |
| PFUnDA | 563.0 | 519.0 | 72 | 4 | Quant | ^13^C_2_ PFUnDA |
| PFUnDA | 563.0 | 269.0 | 72 | 12 | Qual |  |
| ^13^C_2_ PFUnDA | 565.1 | 519.9 | 71 | 4 | IS |  |
| PFDoA | 613.0 | 568.9 | 121 | 4 | Quant | ^13^C_2_ PFDoA |
| PFDoA | 613.0 | 168.9 | 121 | 24 | Qual |  |
| ^13^C_2_ PFDoA | 615.1 | 569.9 | 121 | 4 | IS |  |
| PFBS | 299.0 | 99.0 | 150 | 35 | Quant |  |
| PFBS | 299.0 | 80.0 | 150 | 35 | Qualifier |  |
| PFHxS | 398.9 | 98.9 | 63 | 40 | Quant | ^18^O_2_ PFHxS |
| PFHxS | 398.9 | 80.0 | 63 | 56 | Qual |  |
| ^18^O_2_ PFHxS | 403.1 | 84.0 | 179 | 52 | IS |  |
| PFHpS | 448.9 | 98.9 | 179 | 40 | Quant | ^13^C_4_ PFOA |
| PFHpS | 448.9 | 80.0 | 179 | 64 | Qual |  |
| PFOS | 498.9 | 98.9 | 63 | 44 | Quant | ^13^C_4_ PFOS |
| PFOS | 498.9 | 80.0 | 63 | 72 | Qual |  |
| ^13^C_4_ PFOS | 503.1 | 80.0 | 186 | 72 | IS |  |
| PFOSA | 497.9 | 77.9 | 179 | 36 | Quant | ^13^C_8_ PFOSA |
| PFOSA | 497.9 | 64.0 | 179 | 168 | Qual |  |
| ^13^C_8_ PFOSA | 506.1 | 78.0 | 121 | 36 | IS |  |

IS – Internal standard, QUAL – Qualifier, QUANT - Quantifier

## Table S 8. MS settings (dynamic MRM) of the OP-PFAS analysis.

| **Compound** | **Precursor ion** | **Product ion** | **Fragmentor** | **Collision E (V)** | **Purpose** | **IS** |
| --- | --- | --- | --- | --- | --- | --- |
| PFHxPA | 398.9 | 78.9 | 157 | 35 | Quant | Cl-PFHxPA |
| PFHxPA | 398.9 | 63.0 | 157 | 92 | Qual |  |
| Cl-PFHxPA | 414.9 | 79.0 | 164 | 36 | IS |  |
| 6:2 PAP | 443.0 | 422.9 | 121 | 8 | Qual |  |
| 6:2 PAP | 443.0 | 97.0 | 121 | 12 | Quant | ^13^C-6:2PAP |
| ^13^C-6:2 PAP | 445.0 | 97.0 | 125 | 12 | IS |  |
| PFOPA | 498.9 | 79.0 | 199 | 44 | Quant | Cl-PFHxPA |
| 8:2 PAP | 543.0 | 522.9 | 122 | 8 | Qual |  |
| 8:2 PAP | 543.0 | 97.0 | 122 | 16 | Quant | ^13^C-8:2PAP |
| ^13^C-8:2 PAP | 545.0 | 97.0 | 121 | 24 | IS |  |
| PFDPA | 598.9 | 79.0 | 135 | 56 | Quant | Cl-PFHxPA |
| 6:6 PFPIA | 701.0 | 401.0 | 119 | 56 | Quant | ^13^C-6:2diPAP |
| 6:6 PFPIA | 701.0 | 101.0 | 119 | 88 | Qual |  |
| 6:6 PFPIA | 701.0 | 63.0 | 119 | 92 | Qual |  |
| 6:2 diPAP | 789.0 | 443.0 | 162 | 16 | Qual |  |
| 6:2 diPAP | 789.0 | 97.0 | 162 | 36 | Quant | ^13^C-6:2diPAP |
| ^13^C-6:2 diPAP | 793.0 | 445.0 | 160 | 16 | IS |  |
| ^13^C-6:2 diPAP | 793.0 | 97.0 | 160 | 36 | IS |  |
| 6:8 PFPIA | 801.0 | 500.9 | 199 | 68 | Qual |  |
| 6:8 PFPIA | 801.0 | 401.0 | 199 | 64 | Quant | ^13^C-6:2diPAP |
| 8:8 PFPIA | 901.0 | 500.9 | 196 | 76 | Quant | ^13^C-6:2diPAP |
| 8:8 PFPIA | 901.0 | 63.0 | 196 | 100 | Qual |  |
| 8:2 diPAP | 989.0 | 543.0 | 195 | 24 | Qual |  |
| 8:2 diPAP | 989.0 | 97.0 | 195 | 44 | Quant | ^13^C-6:2diPAP |
| ^13^C-8:2 diPAP | 993.0 | 545.0 | 201 | 24 | IS |  |
| ^13^C-8:2 diPAP | 993.0 | 97.0 | 201 | 44 | IS |  |

IS – Internal standard, QUAL – Qualifier, QUANT - Quantifier

# Quality Assurance/Quality Control

## Table S 9. The accuracy (recovery of native, %) and relative standard deviation of the PFSA, PFCA, and FOSA analysis of enriched (3 ng/mL) bovine serum (n=8) analyzed together with the cat serum samples.

| **Compound** | **Recovery (%)** | **RSD (%)** |
| --- | --- | --- |
| PFHpA | 86 | 9 |
| PFOA | 96 | 4 |
| PFNA | 104 | 15 |
| PFDA | 109 | 13 |
| PFUnDA | 114 | 6 |
| PFDoA | 96 | 13 |
| PFHxS | 89 | 14 |
| PFHpS | 104 | 14 |
| Tot-PFOS* | 107 | 11 |
| FOSA | 97 | 15 |

* Linear and branched PFOS

## Figure S 1. Average recovery (%) of fortified native OP-PFASs in bovine serum samples in three tests and at two levels (duplicates per test).

Test 1. After fortifying the serum, the sample was directly prepared for measurement, (i.e. adding IS and prepared further). No time to equilibrate. The extract was stored after preparation at 4 – 8°C until measurement.

Test 2. After fortifying the serum, the internal standard was added. It was stored for one night at -20°C. The following day the sample preparation was carried out.

Test 3. After fortifying the serum, the sample was stored for one night at -20°C. The following day the internal standard was added, and the preparation was carried out.

## Table S 10. Average (pg/mL) method limit of quantification (LOQ) of PFASs in blood serum samples, set to 3xLOD or based on average levels + 3xSD of the measured PFAS levels in solvent blank samples.

| **Compound** | **LOQ (pg/mL)** | **Compound** | **LOQ (pg/mL)** |
| --- | --- | --- | --- |
| PFHpA | 100 | 6:8 PFPIA | 0.2 |
| PFOA | 100 | 6:6 PFPIA | 0.2 |
| PFNA | 170 | 8:8 PFPIA | 0.3 |
| PFDA | 100 | 6:2 diPAP | 8.0 |
| PFUnDA | 100 | 8:2 diPAP | 8.0 |
| PFDoA | 100 | 6:2 PAP | 52 |
| PFHxS | 68 | 8:2 PAP | 86 |
| PFHpS | 68 | PFHxPA | 6.0 |
| PFOS | 68 | PFOPA | 9.0 |
| FOSA | 68 | PFDPA | 12 |

## Table S 11. Recovery (%) of the PFAS internal standards in cat serum samples (n=27).

|  | **Average (%)** | **Min (%)** | **Max (%)** | **# <10%** |
| --- | --- | --- | --- | --- |
| Cl-PFHxPA | 8 | 4 | 16 | 19 |
| ^13^C_2_-6:2 PAP | 33 | 3 | 71 | 2 |
| ^13^C_2_-8:2 PAP | 32 | 3 | 61 | 2 |
| ^13^C_4_-6:2 diPAP | 45 | 17 | 78 | 0 |
| ^13^C_4_-8:2 diPAP | 70 | 29 | 113 | 0 |
| ^13^C_4_-PFHpA | 32 | 12 | 87 | 0 |
| ^13^C_4_ PFOA | 33 | 12 | 89 | 0 |
| ^13^C_5_ PFNA | 32 | 12 | 88 | 0 |
| ^13^C_2_ PFDA | 124 | 69 | 215 | 0 |
| ^13^C_2_ PFUnDA | 259 | 109 | 368 | 0 |
| ^13^C_2_ PFDoDA | 162 | 86 | 236 | 0 |
| ^18^O_2_ PFHxS | 85 | 32 | 151 | 0 |
| ^13^C_4_ PFOS | 111 | 55 | 157 | 0 |
| ^13^C_8_ FOSA | 51 | 31 | 67 | 0 |

## Table S 12. Average PFSA, PFCA, and 6:2 FTSA concentration (ng/g dust) and the reproducibility (relative standard deviation, RSD %) in quality control dust sample dust (SRM2585, n=8) analyzed simultaneously with the dust samples. The levels are compared (accuracy, %) to reported reference values by Reiner et al. (2011).

| **Compound** | **Average (ng/g)** | **RSD (%)** | **Acc (%)^1^** |
| --- | --- | --- | --- |
| PFBA | 223 | 11 | 90 |
| PFPeA | 225 | 11 | 99 |
| PFHxA | 314 | 8 | 113 |
| PFHpA | 291 | 8 | 112 |
| PFOA | 573 | 11 | 102 |
| PFNA | 91 | 9 | 90 |
| PFDA | 51 | 8 | 133 |
| PFUnDA | 37 | 11 | 78 |
| PFDoA | 29 | 12 | 78 |
| PFTrA | 24 | 13 | 82 |
| PFTeA | 20 | 17 | 102 |
| PFBS | 20 | 12 | - |
| PFHxS | 1498 | 15 | 107 |
| Tot-PFOS | 1791 | 6 | 79 |
| 6:2 FTSA | 101 | 12 | - |

^1^ Reiner et al. Analytical and Bioanalytical Chemistry, 2015. Vol 407(11):2975-2983

## Table S 13. Average levels (ng/g dust) and the reproducibility (relative standard deviation, RSD) of OP-PFAS in quality control (SRM2585) dust samples (n=8) analyzed in parallel with target dust samples. The SRM2585 dust was fortified with a realistic OP-PFAS profile and the accuracy (recovery %) and reproducibility (RSD) of the fortified dust samples (SRM2585, n=8) is reported.

| **Compound** | **Average (ng/g)** | **RSD (%)** | **Fortified (ng/g)** | **Rec (%)** | **RSD (%)** |
| --- | --- | --- | --- | --- | --- |
| 6:2 PAP | 229 | 9 | 630 | 91 | 4 |
| 8:2 PAP | 167 | 9 | 750 | 91 | 8 |
| 6:6 PFPIA | 0.8 | 11 | 3 | 90 | 12 |
| 6:8 PFPIA | 0.8 | 13 | 3 | 87 | 10 |
| 8:8 PFPIA | 0.1 | 46 | 3 | 89 | 14 |
| 6:2 diPAP | 745 | 3 | 3800 | 96 | 6 |
| 8:2 diPAP | 660 | 4 | 3800 | 93 | 5 |
| PFHxPA | 2.3 | 50 | 4 | 76 | 10 |
| PFOPA | 3.1 | 28 | 4 | 104 | 16 |
| PFDPA | 5.2 | 18 | 4 | 99 | 14 |

## Table S 14. The average (ng/g dust) LOQ based on 3xLOD or average +3xSD of the measured PFAS levels in solvent blank samples (n=10) analyzed in parallel with the samples.

| **Compound** | **LOQ (ng/g)** |
| --- | --- |
| PFBA | 31 |
| PFPeA | 1.3 |
| PFHxA | 2.5 |
| PFHpA | 1.6 |
| PFOA | 4.4 |
| PFNA | 1.7 |
| PFDA | 2.0 |
| PFUnDA | 2.7 |
| PFDoA | 2.1 |
| PFTrA | 1.6 |
| PFTeA | 0.3 |
| PFBS | 0.01 |
| PFHxS | 0.5 |
| Tot-PFOS* | 1.1 |
| 6:2 FTSA | 2.0 |
| 6:2PAP | 7.0 |
| 8:2PAP | 5.0 |
| 6:2diPAP | 0.5 |
| 8:2diPAP | 0.9 |
| 6:6PFPIA | 0.1 |
| 6:8PFPIA | 2.8 |
| 8:8PFPIA | 2.8 |
| PFHxPA | 1.3 |
| PFOPA | 0.5 |
| PFDPA | 0.4 |

* Branched and linear PFOS

# Results in details

## Table S 15. Detailed cat serum sample information on cats weight, age, and gender, as well as total thyroxine (tT4), thyroid stimulating hormone (TSH) and ΣPFAS levels (pg/mL).

| **Family** | **Cat no** | **Weight**  **(kg)** | **Age**  **(year)** | **tT4**  **(nmol/L)** | **TSH**  **(µg/L)** | **Gender** | **Σ PFASs** |
| --- | --- | --- | --- | --- | --- | --- | --- |
| 1 | 1* | 4+4 | 12 | 39 | 0.040 | m/f | 12300 |
| 2 | 4* | 6+14 | 8 | 33 | 0.110 | m/m | 6740 |
| 3 | 5 | 3.5 | 11 | <6 | **0.015** | f | 7270 |
| 3 | 6 | 3.5 | 11 | 9.0 | 0.060 | f | 4730 |
| 4 | 7 | 4.0 | 2.5 | 13 | 0.140 | f^#^ | 2210 |
| 4 | 8 | 6.0 | 1.5 | 13 | 0.120 | m^#^ | 1620 |
| 5 | 9 | 3.5 | 1 | 27 | 0.060 | m | 3180 |
| 5 | 10 | 3.5 | 1 | 27 | 0.040 | m | 3280 |
| 6 | 11 | 4.0 | 3 | 31 | **0.015** | m | 22500 |
| 7 | 12 | 4.0 | 7 | 24 | 0.040 | f | 10100 |
| 7 | 13 | 4.0 | 13 | 31 | **0.015** | m | 4610 |
| 8 | 14 | 5.5 | 2 | 23 | **0.015** | m | 8070 |
| 9 | 15 | 11 | 10 | 35 | 0.030 | m | 6040 |
| 9 | 16 | 3.0 | 2.5 | 25 | 0.040 | f^#^ | 12100 |
| 9 | 17 | 3.0 | 2.5 | 25 | 0.070 | f^#^ | 11100 |
| 9 | 18 | -- | 6 | 25 | **0.015** | f | 11600 |
| 10 | 19 | 3.5 | 4 | 28 | **0.015** | f | 9200 |
| 11 | 20 | 6.0 | 7 | 34 | 0.040 | m | 6100 |
| 12 | 21 | 3.5 | 13 | 25 | 0.060 | f | 8240 |
| 13 | 22 | 6.5 | 6.5 | 26 | 0.030 | m | 2330 |
| 14 | 23 | 4.5 | 2 | 23 | 0.120 | m | 5310 |
| 14 | 24 | 3.5 | 2 | 34 | 0.200 | f | 6020 |
| 15 | 25 | 5.0 | 10 | 22 | 0.090 | m^#^ | 9810 |
| 16 | 26 | 4.5 | 9 | 32 | 0.080 | m | 12100 |
| 17 | 27 | 3.0 | 8 | 27 | 0.140 | f^#^ | 6410 |
| 17 | 28 | 8.0 | 8 | 43 | 0.060 | m | 6320 |
| 17 | 29 | 6.0 | 5 | 25 | 0.070 | m | 5170 |
|  | Mean | 4.7 | 6.2 | 27 | 0.064 | 59% m | 7580 |
|  | Median | 4.0 | 6.5 | 27 | 0.060 | 41% f | 6410 |
|  | Min | 3.0 | 1 | 9 | 0.015 |  | 1620 |
|  | Max | 14 | 13 | 43 | 0.200 |  | 22500 |

* Pooled serum from cat siblings. Bold TSH numbers indicate LOQ. ^#^ non-castrated males (m) and females (f).

## Table S 16. Detailed PFCA and PFSA levels (pg/mL) determined in cat serum samples.

| **Family** | **Cat no** | **PFHpA** | **PFOA** | **PFNA** | **PFDA** | **PFUnDA** | **PFDoDA** | **PFHxS** | **PFHpS** | **PFOS** | **Σ PFCAs/SAs** |
| --- | --- | --- | --- | --- | --- | --- | --- | --- | --- | --- | --- |
| 1 | 1 | 3750 | 3543 | **167** | 875 | 782 | 250 | 219 | 167 | 2397 | 12149 |
| 2 | 4 | 1350 | 802 | 198 | 156 | 240 | **63** | 1146 | 104 | 2605 | 6665 |
| 3 | 5 | 1140 | 1355 | 198 | 354 | 323 | 115 | 792 | <LOD | 2813 | 7090 |
| 3 | 6 | 140 | 636 | **135** | 208 | 229 | **94** | 531 | <LOD | 2709 | 4683 |
| 4 | 7 | 313 | 531 | **94** | 146 | 208 | **63** | **21** | <LOD | 834 | 2210 |
| 4 | 8 | **70** | 396 | <LOD | 167 | **94** | **83** | 125 | <LOD | 677 | 1612 |
| 5 | 9 | 440 | 740 | **94** | 125 | 135 | **21** | 219 | 42 | 1355 | 3170 |
| 5 | 10 | **70** | 479 | **156** | 250 | 271 | **31** | 521 | <LOD | 1355 | 3133 |
| 6 | 11 | 1560 | 14588 | 438 | 438 | 1459 | 177 | 396 | 156 | 3230 | 22442 |
| 7 | 12 | 720 | 531 | 854 | 865 | 907 | 271 | 854 | **31** | 5002 | 10035 |
| 7 | 13 | 820 | 490 | 313 | 302 | 584 | 115 | <LOD | <LOD | 1980 | 4602 |
| 8 | 14 | 580 | 2188 | 938 | 688 | 709 | 135 | 584 | **52** | 2188 | 8061 |
| 9 | 15 | **230** | 688 | 479 | 594 | 1032 | 229 | 458 | <LOD | 2292 | 6003 |
| 9 | 16 | 3230 | 1980 | 771 | 1146 | 1459 | 292 | 281 | <LOD | 2605 | 11764 |
| 9 | 17 | 2600 | 2397 | 750 | 1459 | 1667 | 281 | 396 | <LOD | 1563 | 11113 |
| 9 | 18 | 4060 | 1563 | 1042 | 1042 | 1355 | 271 | 208 | <LOD | 2084 | 11625 |
| 10 | 19 | 1670 | 1146 | **146** | 573 | 354 | 188 | 1876 | 115 | 3126 | 9193 |
| 11 | 20 | **313** | 1667 | 240 | 386 | 594 | 115 | 521 | 73 | 2188 | 6096 |
| 12 | 21 | 1980 | 1011 | **146** | 198 | 313 | 73 | 2084 | <LOD | 2397 | 8201 |
| 13 | 22 | **400** | **198** | 188 | 240 | 177 | <LOD | 229 | <LOD | 875 | 2307 |
| 14 | 23 | <LOD | 1563 | **104** | 198 | 427 | 52 | 573 | <LOD | 2397 | 5314 |
| 14 | 24 | 500 | 1042 | 219 | 333 | 761 | 104 | 750 | <LOD | 2292 | 6002 |
| 15 | 25 | 950 | 2292 | 375 | 458 | 865 | 115 | 1021 | **63** | 3647 | 9787 |
| 16 | 26 | 4270 | 1980 | 667 | 615 | 490 | 94 | 604 | **42** | 3334 | 12096 |
| 17 | 27 | **170** | 354 | <LOD | 427 | 438 | **240** | 261 | **135** | 4376 | 6401 |
| 17 | 28 | 320 | 2397 | 688 | 761 | 573 | 188 | 146 | <LOD | 1250 | 6322 |
| 17 | 29 | 220 | 2084 | 375 | 427 | 292 | **94** | 323 | <LOD | 1355 | 5170 |
| n>LOD | | 96% | 100% | 93% | 100% | 100% | 96% | 96% | 41% | 100% |  |
| n>LOQ | | 78% | 96% | 70% | 100% | 96% | 70% | 93% | 22% | 100% |  |
| Mean |  | 1180 | 1802 | 362 | 497 | 620 | 139 | 561 | 36 | 2331 | 7528 |
| Median | | 580 | 1146 | 219 | 427 | 490 | 115 | 458 | 0 | 2292 | 6401 |
| Min |  | <LOD | <LOQ | <LOD | 125 | <LOQ | <LOD | <LOD | <LOD | 677 | 1612 |
| Max |  | 4270 | 14588 | 1042 | 1459 | 1667 | 292 | 2084 | 167 | 5002 | 22442 |

Bold numbers indicate levels <LOQ. n.a. not analysed due to lost sample.

## Table S 17. Detailed FOSA and organophosphorus PFAS levels (pg/mL) determined in cat serum samples.

| **Family** | **Cat no** | **FOSA** | **6:2PAP** | **8:2PAP** | **6:2diPAP** | **8:2diPAP** | **6:8PFPIA** | **6:6PFPIA** | **8:8PFPIA** | **Σ OP-PFASs^1^** |
| --- | --- | --- | --- | --- | --- | --- | --- | --- | --- | --- |
| 1 | 1 | **31** | 92 | <LOD | 11 | 18 | 9.1 | <LOD | <LOD | 130 |
| 2 | 4 | <LOD | <LOD | <LOD | 37 | 39 | <LOD | <LOD | <LOD | 76 |
| 3 | 5 | 104.2 | **37** | <LOD | 10 | **1.9** | 17 | 5.2 | 0.82 | 72 |
| 3 | 6 | <LOD | <LOD | <LOD | 15 | 26 | 6.5 | 0.4 | 1 | 49 |
| 4 | 7 | <LOD | <LOD | <LOD | <LOD | **1.5** | <LOD | <LOD | <LOD | 2 |
| 4 | 8 | <LOD | <LOD | <LOD | <LOD | **3.8** | <LOD | <LOD | <LOD | 4 |
| 5 | 9 | <LOD | <LOD | <LOD | <LOD | **4.8** | <LOD | <LOD | <LOD | 5 |
| 5 | 10 | <LOD | 38 | 75 | 7.7 | 20 | 6.8 | <LOD | <LOD | 148 |
| 6 | 11 | <LOD | 53 | <LOD | 6.2 | **3.4** | <LOD | <LOD | <LOD | 63 |
| 7 | 12 | <LOD | <LOD | <LOD | <LOD | 8.9 | 3.5 | 3.3 | 1.1 | 17 |
| 7 | 13 | <LOD | <LOD | <LOD | **5.3** | **4.2** | <LOD | <LOD | <LOD | 10 |
| 8 | 14 | <LOD | <LOD | <LOD | 8 | **4.1** | <LOD | <LOD | <LOD | 12 |
| 9 | 15 | <LOD | **19** | <LOD | 8.6 | 12 | <LOD | <LOD | <LOD | 40 |
| 9 | 16 | <LOD | 126 | **46** | 127 | 62 | <LOD | <LOD | <LOD | 361 |
| 9 | 17 | <LOD | <LOD | <LOD | <LOD | 12 | 0.88 | <LOD | <LOD | 13 |
| 9 | 18 | <LOD | <LOD | <LOD | **2.5** | 13 | <LOD | <LOD | <LOD | 16 |
| 10 | 19 | <LOD | <LOD | <LOD | **1.4** | **4.4** | <LOD | 3.6 | <LOD | 9 |
| 11 | 20 | <LOD | <LOD | <LOD | <LOD | <LOD | <LOD | <LOD | <LOD | 0 |
| 12 | 21 | <LOD | <LOD | <LOD | **7.2** | 35 | <LOD | <LOD | <LOD | 42 |
| 13 | 22 | <LOD | <LOD | <LOD | 11 | **8.7** | <LOD | <LOD | <LOD | 20 |
| 14 | 23 | <LOD | <LOD | <LOD | <LOD | <LOD | <LOD | <LOD | <LOD | 0 |
| 14 | 24 | <LOD | <LOD | <LOD | <LOD | 18 | <LOD | <LOD | <LOD | 18 |
| 15 | 25 | <LOD | <LOD | <LOD | **4.1** | 21 | <LOD | <LOD | <LOD | 25 |
| 16 | 26 | <LOD | <LOD | <LOD | **2.4** | **2** | 12 | <LOD | <LOD | 16 |
| 17 | 27 | <LOD | <LOD | <LOD | **6.3** | **5.2** | <LOD | <LOD | <LOD | 12 |
| 17 | 28 | <LOD | n.a. | n.a. | n.a. | n.a. | n.a. | n.a. | n.a. | 0 |
| 17 | 29 | <LOD | n.a. | n.a. | n.a. | n.a. | n.a. | n.a. | n.a. | 0 |
| n>LOD | | 7% | 24% | 8% | 68% | 92% | 28% | 16% | 12% |  |
| n>LOQ | | 4% | 16% | 4% | 40% | 48% | 28% | 16% | 12% |  |
| Mean |  | 5.0 | 15 | 4.8 | 11 | 13 | 2.2 | 0.5 | 0.1 | 43 |
| Median | | 0,0 | 0.0 | 0.0 | 5.3 | 8.7 | 0.0 | 0.0 | 0.0 | 16 |
| Min |  | <LOD | <LOD | <LOD | <LOD | <LOD | <LOD | <LOD | <LOD | 0 |
| Max |  | 104 | 126 | 75 | 127 | 62 | 17 | 5.2 | 1.1 | 361 |

^1^ Sum of organophosphorus PFAS, not including FOSA.

## Table S 18. PFCA and PFCA concentrations (ng/g dust) determined in dust samples from the living rooms.

| **Family** | **PFBA** | **PFPeA** | **PFHxA** | **PFHpA** | **PFOA** | **PFNA** | **PFDA** | **PFUnDA** | **PFDoA** | **PFTrA** | **PFTeA** | **PFBS** | **PFHxS** | **PFOS** | **ΣPFCAs/SAs** |
| --- | --- | --- | --- | --- | --- | --- | --- | --- | --- | --- | --- | --- | --- | --- | --- |
| 1 | <LOD | <LOD | 44 | 7.2 | 33 | 7.6 | 15 | **1.9** | 8.0 | <LOD | **2.2** | <LOD | 3.5 | 13 | 135 |
| 2 | <LOD | <LOD | <LOD | <LOD | **2.6** | 3.5 | **1.3** | <LOD | <LOD | <LOD | <LOD | <LOD | <LOD | <LOD | 7 |
| 3 | <LOD | <LOD | 7.3 | **2.3** | 9.7 | **2.4** | 3.6 | <LOD | <LOD | <LOD | <LOD | <LOD | <LOD | 7.9 | 33 |
| 4 | <LOD | <LOD | **4.6** | <LOD | 5.0 | **1.2** | **2.4** | <LOD | <LOD | <LOD | <LOD | <LOD | <LOD | 7.4 | 21 |
| 5 | <LOD | <LOD | 7.9 | **4.2** | 10 | 3.1 | 3.2 | **1.2** | **2.2** | <LOD | <LOD | <LOD | **1.3** | 23 | 56 |
| 6 | <LOD | <LOD | 11 | 10 | 260 | 26 | 100 | 22 | 110 | 9.1 | 56 | <LOD | **1.5** | 19 | 625 |
| 7 | <LOD | <LOD | **2.1** | <LOD | 5.4 | 4.4 | 3.3 | **1.4** | <LOD | <LOD | <LOD | <LOD | <LOD | 24 | 41 |
| 8 | <LOD | <LOD | 6.1 | **2.0** | 7.0 | 7.4 | **2.9** | **1.2** | <LOD | <LOD | <LOD | <LOD | <LOD | 6.7 | 33 |
| 9 | <LOD | <LOD | **3.1** | <LOD | 4.8 | **2.2** | **1.6** | **1.0** | <LOD | <LOD | <LOD | <LOD | <LOD | 13 | 26 |
| 10 | **11** | <LOD | **4.8** | <LOD | 5.5 | **2.3** | 4.1 | **0.9** | **2.7** | <LOD | **1.8** | <LOD | <LOD | 15 | 48 |
| 11 | <LOD | <LOD | 17 | 11 | 59 | 24 | 22 | 6.8 | 7.9 | **3.0** | 6.8 | <LOD | <LOD | 60 | 218 |
| 12 | <LOD | <LOD | **5.5** | **2.4** | 5.7 | **1.2** | 3.2 | <LOD | <LOD | <LOD | <LOD | <LOD | <LOD | 24 | 42 |
| 13 |  |  |  |  |  |  |  |  |  |  |  |  |  |  |  |
| 14 |  |  |  |  |  |  |  |  |  |  |  |  |  |  |  |
| 15 | <LOD | <LOD | 8.0 | **2.7** | 8.3 | 3.4 | 3.4 | <LOD | <LOD | **2.0** | <LOD | <LOD | <LOD | 13 | 41 |
| 16 | <LOD | <LOD | **5.8** | <LOD | 5.3 | **2.3** | **1.9** | **1.0** | <LOD | <LOD | <LOD | <LOD | <LOD | 3.9 | 20 |
| 17 |  |  |  |  |  |  |  |  |  |  |  |  |  |  |  |
| n>LOD | 7% | 0% | 93% | 57% | 100% | 100% | 100% | 64% | 36% | 21% | 29% | 0% | 21% | 100% |  |
| n>LOQ | 0% | 0% | 50% | 21% | 93% | 57% | 64% | 14% | 21% | 7% | 14% | 0% | 7% | 93% |  |
| Mean | 0.8 |  | 9.1 | 3.0 | 30 | 6.5 | 12 | 2.7 | 9.3 | 1.0 | 4.8 |  | 0.5 | 16 | 96 |
| Median | 0.0 |  | 6.0 | 2.2 | 6.4 | 3.3 | 3.3 | 1.0 | 0.0 | 0.0 | 0.0 |  | 0.0 | 13 | 41 |
| Min | <LOD |  | <LOD | <LOD | <LOQ | <LOQ | <LOQ | <LOD | <LOD | <LOD | <LOD |  | <LOD | <LOD | 7 |
| Max | 11 |  | 44 | 11 | 260 | 26 | 100 | 22 | 110 | 9 | 56 |  | 4 | 60 | 625 |
| SD | 2.9 |  | 11 | 3.8 | 68 | 8.1 | 26 | 5.8 | 29 | 2.5 | 15 |  | 1.0 | 15 | 162 |

## Table S 19. 6:2 FTSA and organophosphorus PFAS concentrations and ΣPFASs (ng/g dust) determined in dust samples from the living rooms.

| **Family** | **6:2 FTSA** | **6:2PAP** | **8:2PAP** | **6:6PFPIA** | **6:8PFPIA** | **8:8PFPIA** | **6:2diPAP** | **8:2diPAP** | **PFHxPA** | **PFOPA** | **PFDPA** | **ΣOP-PFASs^1^** | **ΣPFASs^2^** |
| --- | --- | --- | --- | --- | --- | --- | --- | --- | --- | --- | --- | --- | --- |
| 1 | **2.8** | 13 | 9.1 | 1.1 | 1.8 | 0.7 | 18 | 14 | 6.9 | 13 | 24 | 102 | 240 |
| 2 | <LOD | 459 | 364 | <LOD | <LOD | <LOD | 598 | 552 | **3.4** | **1.5** | <LOD | 1978 | 1985 |
| 3 | 11 | 7.6 | 12 | 4.3 | 7.3 | 2.7 | 13 | 7.4 | 13 | 63 | 63 | 193 | 238 |
| 4 | <LOD | 36 | 27 | <LOD | <LOD | <LOD | 58 | 43 | <LOD | **1.8** | <LOD | 166 | 186 |
| 5 | **1.2** | 18 | 15 | <LOD | <LOD | **0.1** | 64 | 33 | <LOD | **2.1** | <LOD | 132 | 190 |
| 6 | 22 | 28 | 24 | <LOD | **0.5** | **0.2** | 48 | 51 | **2.3** | **3.7** | 6.2 | 164 | 811 |
| 7 | **1.8** | 12 | 19 | <LOD | <LOD | **0.1** | 28 | 56 | **2.6** | <LOD | 6.1 | 124 | 166 |
| 8 | **1.2** | 47 | 37 | 16 | 27 | 11 | 71 | 59 | 31 | 1767 | 70 | 2136 | 2171 |
| 9 | <LOD | 16 | 13 | <LOD | <LOD | <LOD | 46 | 39 | **2.2** | **2.6** | <LOD | 119 | 145 |
| 10 | **1.6** | 394 | 191 | 3.5 | 7.5 | 2.7 | 470 | 277 | 6.1 | 18 | 17 | 1387 | 1436 |
| 11 | 23 | 36 | 28 | <LOD | **0.4** | **0.1** | 160 | 82 | <LOD | 4.1 | **5.6** | 316 | 557 |
| 12 | <LOD | 115 | 94 | <LOD | <LOD | **0.1** | 197 | 192 | <LOD | 9.1 | **3.9** | 611 | 653 |
| 13 |  |  |  |  |  |  |  |  |  |  |  |  |  |
| 14 |  |  |  |  |  |  |  |  |  |  |  |  |  |
| 15 | 3.4 | 71 | 46 | <LOD | <LOD | <LOD | 139 | 96 | <LOD | <LOD | <LOD | 352 | 396 |
| 16 | **2.6** | 17 | 8.0 | <LOD | <LOD | <LOD | 39 | 17 | <LOD | <LOD | <LOD | 81 | 104 |
| 17 |  |  |  |  |  |  |  |  |  |  |  |  |  |
| n>LOD | 71% | 100% | 100% | 29% | 43% | 64% | 100% | 100% | 57% | 79% | 57% |  |  |
| n>LOQ | 29% | 100% | 100% | 29% | 29% | 29% | 100% | 100% | 29% | 43% | 43% |  |  |
| Mean | 5.0 | 91 | 63 | 1.8 | 3.2 | 1.3 | 139 | 108 | 4.8 | 135 | 14 | 561 | 663 |
| Median | 1.7 | 32 | 26 | 0.0 | 0.0 | 0.1 | 61 | 54 | 2.3 | 3.2 | 4.8 | 180 | 318 |
| Min | <LOD | 7.6 | 8.0 | <LOD | <LOD | <LOD | 13 | 7.4 | <LOD | <LOD | <LOD | 81 | 104 |
| Max | 23 | 459 | 364 | 16 | 27 | 11 | 598 | 552 | 31 | 1767 | 70 | 2136 | 2171 |
| SD | 7.9 | 146 | 99 | 4.3 | 7.3 | 3.0 | 178 | 148 | 8.4 | 470 | 23 | 720 | 700 |

^1^ Sum of organophosphorus PFASs, not including 6:2 FTSA. ^2^ Sum of all analyzed PFASs.

## Table S 20. PFCA and PFCA concentrations (ng/g dust) determined in dust samples from the adult bedrooms.

| **Family** | **PFBA** | **PFPeA** | **PFHxA** | **PFHpA** | **PFOA** | **PFNA** | **PFDA** | **PFUnDA** | **PFDoA** | **PFTrA** | **PFTeA** | **PFBS** | **PFHxS** | **PFOS** | **ΣPFCAs/SAs** |
| --- | --- | --- | --- | --- | --- | --- | --- | --- | --- | --- | --- | --- | --- | --- | --- |
| 1 | **17** | <LOD | 28 | **2.6** | 20 | 4.6 | 13 | **1.9** | **6.4** | <LOD | **4.9** | <LOD | <LOD | **3.2** | 102 |
| 2 | <LOD | <LOD | 8.5 | **3.7** | 17 | 6.3 | 5.0 | **1.4** | **2.6** | <LOD | <LOD | <LOD | <LOD | 20 | 65 |
| 3 | <LOD | <LOD | **4.8** | <LOD | 10 | 3.3 | 3.4 | <LOD | **1.8** | <LOD | <LOD | <LOD | <LOD | 15 | 38 |
| 4 | <LOD | <LOD | <LOD | <LOD | 2.8 | <LOD | <LOD | <LOD | <LOD | <LOD | <LOD | <LOD | <LOD | 6.5 | 9 |
| 5 | <LOD | <LOD | 7.5 | 6.6 | 6.4 | **2.5** | **1.1** | **1.0** | <LOD | <LOD | <LOD | <LOD | <LOD | 8.3 | 33 |
| 6 | <LOD | <LOD | 12 | **4.2** | 230 | 6.1 | 24 | 3.4 | 21 | **2.5** | 17 | <LOD | <LOD | 16 | 336 |
| 7 | <LOD | <LOD | 27 | 12 | 23 | 14 | 16 | 6.5 | 9.7 | **4.8** | 6.7 | <LOD | <LOD | 4.9 | 125 |
| 8 | <LOD | <LOD | 11 | <LOD | 12 | 9.1 | 3.7 | **1.3** | **1.8** | <LOD | <LOD | <LOD | **1.1** | 9.8 | 50 |
| 9 |  |  |  |  |  |  |  |  |  |  |  |  |  |  |  |
| 10 | <LOD | <LOD | **5.0** | **2.3** | 7.1 | **1.0** | **2.1** | **1.2** | <LOD | <LOD | **1.7** | <LOD | **2.1** | 22 | 45 |
| 11 | **23** | **23** | 99 | 95 | 570 | 44 | 81 | 12 | 46 | 7.1 | 45 | <LOD | **2.3** | 220 | 1267 |
| 12 |  |  |  |  |  |  |  |  |  |  |  |  |  |  |  |
| 13 | <LOD | <LOD | **2.4** | <LOD | **3.5** | 4.3 | **2.3** | <LOD | <LOD | <LOD | <LOD | <LOD | <LOD | 10 | 23 |
| 14 | <LOD | <LOD | **6.2** | <LOD | 4.0 | **1.7** | **1.1** | <LOD | <LOD | <LOD | <LOD | <LOD | <LOD | 3.5 | 17 |
| 15 | <LOD | <LOD | **4.8** | <LOD | 5.4 | **1.8** | **2.3** | <LOD | <LOD | <LOD | <LOD | <LOD | <LOD | 7.4 | 22 |
| 16 |  |  |  |  |  |  |  |  |  |  |  |  |  |  |  |
| 17 | <LOD | <LOD | **2.8** | <LOD | 9.8 | <LOD | **1.2** | <LOD | <LOD | <LOD | <LOD | <LOD | <LOD | 22 | 36 |
| n>LOD | 14% | 7% | 93% | 50% | 100% | 86% | 93% | 57% | 50% | 21% | 36% | 0% | 21% | 100% |  |
| n>LOQ | 0% | 0% | 50% | 21% | 93% | 57% | 50% | 21% | 21% | 7% | 21% | 0% | 0% | 93% |  |
| Mean | 2.9 | 1.6 | 16 | 9.0 | 66 | 7.1 | 11 | 2.1 | 6.4 | 1.0 | 5.4 |  | 0.4 | 26 | 155 |
| Median | 0.0 | 0.0 | 6.9 | 1.2 | 9.9 | 3.8 | 2.9 | 1.1 | 0.9 | 0.0 | 0.0 |  | 0.0 | 9.9 | 41 |
| Min | <LOD | <LOD | <LOD | <LOD | <LOQ | <LOD | <LOD | <LOD | <LOD | <LOD | <LOD |  | <LOD | <LOQ | 9 |
| Max | 23 | 23 | 99 | 95 | 570 | 44 | 81 | 12 | 46 | 7.1 | 45 |  | 2.3 | 220 | 1267 |
| SD | 7.4 | 6.1 | 25 | 25 | 157 | 11 | 21 | 3.4 | 13 | 2.2 | 12 |  | 0.8 | 56 | 331 |

## Table S 21. 6:2 FTSA and organophosphorus PFASs concentrations (ng/g dust) determined in dust samples from the adult bedrooms.

| **Family** | **6:2 FTSA** | **6:2PAP** | **8:2PAP** | **6:6PFPIA** | **6:8PFPIA** | **8:8PFPIA** | **6:2diPAP** | **8:2diPAP** | **PFHxPA** | **PFOPA** | **PFDPA** | **ΣOP-PFASs^1^** | **ΣPFASs^2^** |
| --- | --- | --- | --- | --- | --- | --- | --- | --- | --- | --- | --- | --- | --- |
| 1 | <LOD | **5.2** | 5.8 | **0.4** | **0.6** | **0.3** | 9.3 | 7.1 | **2.1** | 12 | **6.5** | 49 | 151 |
| 2 | <LOD | 72 | 66 | <LOD | <LOD | <LOD | 117 | 112 | <LOD | **2.1** | <LOD | 369 | 434 |
| 3 | 3.1 | **3.2** | 6.1 | 5.1 | 6.8 | 2.7 | 8.0 | 6.3 | 18 | 59 | 62 | 177 | 219 |
| 4 | <LOD | 27 | 18 | <LOD | <LOD | <LOD | 103 | 74 | **1.8** | <LOD | <LOD | 224 | 233 |
| 5 | <LOD | 13 | 8.2 | <LOD | <LOD | <LOD | 67 | 26 | <LOD | <LOD | **2.1** | 116 | 150 |
| 6 | **1.3** | 15 | 10 | **0.3** | **0.3** | **0.3** | 23 | 23 | **4.0** | 4.8 | **3.8** | 85 | 422 |
| 7 | **1.8** | 153 | 74 | <LOD | **0.5** | **0.3** | 306 | 312 | **1.9** | 7.6 | **2.6** | 858 | 984 |
| 8 | <LOD | 43 | 40 | 1.5 | 2.4 | 0.9 | 65 | 53 | 20 | 5.8 | <LOD | 232 | 281 |
| 9 |  |  |  |  |  |  |  |  |  |  |  |  |  |
| 10 | <LOD | 110 | 53 | 1.3 | 2.7 | 1.1 | 169 | 74 | <LOD | **2.0** | <LOD | 413 | 458 |
| 11 | 57 | 29 | 26 | **0.5** | 1.4 | 0.8 | 117 | 64 | <LOD | 9.0 | <LOD | 248 | 1572 |
| 12 |  |  |  |  |  |  |  |  |  |  |  |  |  |
| 13 | **1.2** | 34 | 13 | <LOD | <LOD | <LOD | 52 | 30 | <LOD | <LOD | <LOD | 129 | 153 |
| 14 | 41 | 9.9 | 6.9 | <LOD | <LOD | <LOD | 12 | 8.9 | <LOD | <LOD | <LOD | 38 | 95 |
| 15 | 3.0 | 18 | 14 | <LOD | <LOD | <LOD | 65 | 44 | <LOD | **1.7** | <LOD | 143 | 167 |
| 16 |  |  |  |  |  |  |  |  |  |  |  |  |  |
| 17 | <LOD | 224 | 179 | <LOD | <LOD | <LOD | 338 | 295 | <LOD | <LOD | <LOD | 1036 | 1072 |
| n>LOD | 50% | 100% | 100% | 43% | 50% | 50% | 100% | 100% | 43% | 64% | 36% |  |  |
| n>LOQ | 29% | 86% | 100% | 21% | 29% | 29% | 100% | 100% | 14% | 43% | 7% |  |  |
| Mean | 7.7 | 54 | 37 | 0.7 | 1.1 | 0.5 | 104 | 81 | 3.4 | 7.4 | 5.5 | 294 | 456 |
| Median | 0.6 | 28 | 16 | 0.0 | 0.2 | 0.2 | 66 | 49 | 0.0 | 2.1 | 0.0 | 201 | 257 |
| Min | <LOD | <LOQ | 5.8 | <LOD | <LOD | <LOD | 8.0 | 6.3 | <LOD | <LOD | <LOD | 38 | 95 |
| Max | 57 | 224 | 179 | 5.1 | 6.8 | 2.7 | 338 | 312 | 20 | 59 | 62 | 1036 | 1572 |
| SD | 18 | 65 | 47 | 1.4 | 1.9 | 0.7 | 104 | 99 | 6.7 | 15 | 16 | 299 | 442 |

^1^ Sum of organophosphorus PFASs, not including 6:2 FTSA. ^2^ Sum of all analyzed PFASs.

## Table S 22. PFCA and PFCA concentrations (ng/g dust) determined in dust samples from the child rooms.

| **Family** | **PFBA** | **PFPeA** | **PFHxA** | **PFHpA** | **PFOA** | **PFNA** | **PFDA** | **PFUnDA** | **PFDoA** | **PFTrA** | **PFTeA** | **PFBS** | **PFHxS** | **PFOS** | **ΣPFCAs/SAs** |
| --- | --- | --- | --- | --- | --- | --- | --- | --- | --- | --- | --- | --- | --- | --- | --- |
| 1 | <LOD | <LOD | 25 | **3.7** | 21 | 4.3 | 13 | **1.5** | 8.7 | **1.8** | 8.9 | <LOD | <LOD | **1.3** | 89 |
| 2 | <LOD | <LOD | **2.5** | **2.0** | 7.0 | 3.4 | **2.0** | <LOD | <LOD | <LOD | <LOD | <LOD | <LOD | 20 | 37 |
| 3 |  |  |  |  |  |  |  |  |  |  |  |  |  |  |  |
| 4 |  |  |  |  |  |  |  |  |  |  |  |  |  |  |  |
| 5 | <LOD | <LOD | 6.6 | **2.0** | 11 | **2.1** | **2.6** | <LOD | <LOD | <LOD | <LOD | <LOD | <LOD | 18 | 42 |
| 6 | <LOD | <LOD | 6.7 | <LOD | 47 | 3.5 | 7.3 | **1.4** | **5.7** | <LOD | **3.8** | <LOD | <LOD | 7.9 | 83 |
| 7 | <LOD | <LOD | **2.3** | <LOD | 5.5 | **2.9** | **2.4** | **1.6** | <LOD | <LOD | <LOD | <LOD | <LOD | 10 | 25 |
| 8 | <LOD | <LOD | **4.5** | **2.7** | 17 | 13 | 11 | **2.6** | 10 | <LOD | <LOD | <LOD | <LOD | 41 | 102 |
| 9 |  |  |  |  |  |  |  |  |  |  |  |  |  |  |  |
| 10 | <LOD | <LOD | **3.6** | **2.5** | 7.2 | **1.8** | **1.9** | <LOD | <LOD | <LOD | <LOD | <LOD | **1.2** | 20 | 38 |
| 11 | <LOD | <LOD | 32 | 9.7 | 64 | 15 | 21 | 4.6 | 11 | **3.6** | 8.4 | <LOD | **1.4** | 73 | 244 |
| 12 | <LOD | <LOD | 21 | 7.4 | 24 | **1.8** | **2.9** | <LOD | <LOD | <LOD | <LOD | <LOD | 4.1 | 48 | 109 |
| 13 | <LOD | <LOD | **2.4** | <LOD | **1.9** | 7.1 | <LOD | <LOD | <LOD | <LOD | <LOD | <LOD | <LOD | 15.0 | 26 |
| 14 |  |  |  |  |  |  |  |  |  |  |  |  |  |  |  |
| 15 | <LOD | <LOD | 9.1 | **2.3** | 7.8 | **2.6** | 3.4 | <LOD | <LOD | <LOD | <LOD | <LOD | <LOD | 8.7 | 34 |
| 16 | <LOD | <LOD | **3.3** | <LOD | 6.8 | <LOD | **1.6** | <LOD | <LOD | <LOD | <LOD | <LOD | <LOD | 4.8 | 17 |
| 17 | <LOD | <LOD | **2.2** | <LOD | 12.0 | **1.2** | **1.5** | <LOD | <LOD | <LOD | <LOD | <LOD | <LOD | 6.6 | 24 |
| n>LOD | 0% | 0% | 100% | 62% | 100% | 92% | 92% | 38% | 31% | 15% | 23% | 0% | 23% | 100% |  |
| n>LOQ | 0% | 0% | 46% | 15% | 92% | 46% | 38% | 8% | 23% | 0% | 15% | 0% | 8% | 92% |  |
| Mean |  |  | 9.3 | 2.5 | 18 | 4.5 | 5.4 | 0.9 | 2.7 | 0.4 | 1.6 |  | 0.5 | 21 | 67 |
| Median |  |  | 4.5 | 2.0 | 11 | 2.9 | 2.6 | 0.0 | 0.0 | 0.0 | 0.0 |  | 0.0 | 15 | 38 |
| Min |  |  | <LOQ | <LOD | <LOQ | <LOD | <LOD | <LOD | <LOD | <LOD | <LOD |  | <LOD | <LOQ | 17 |
| Max |  |  | 32 | 10 | 64 | 15 | 21 | 4.6 | 11 | 3.6 | 8.9 |  | 4.1 | 73 | 244 |
| SD |  |  | 10 | 3.0 | 18 | 4.6 | 6.1 | 1.4 | 4.4 | 1.1 | 3.3 |  | 1.2 | 21 | 62 |

## Table S 23. FTSA and organophosphorus PFASs concentrations (ng/g dust) determined in dust samples from the child rooms.

| **Family** | **6:2 FTSA** | **6:2PAP** | **8:2PAP** | **6:6PFPIA** | **6:8PFPIA** | **8:8PFPIA** | **6:2diPAP** | **8:2diPAP** | **PFHxPA** | **PFOPA** | **PFDPA** | **ΣOP-PFASs^1^** | **ΣPFASs^2^** |
| --- | --- | --- | --- | --- | --- | --- | --- | --- | --- | --- | --- | --- | --- |
| 1 | **2.6** | 16 | 10 | 3.0 | 4.7 | 1.5 | 32 | 19 | 11 | 37 | 48 | 182 | 274 |
| 2 | <LOD | 171 | 138 | <LOD | <LOD | <LOD | 224 | 208 | <LOD | **2.2** | <LOD | 743 | 780 |
| 3 |  |  |  |  |  |  |  |  |  |  |  |  |  |
| 4 |  |  |  |  |  |  |  |  |  |  |  |  |  |
| 5 | 9.4 | **5.7** | **3.4** | <LOD | <LOD | **0.1** | 19 | 7.6 | <LOD | **4.4** | <LOD | 40 | 92 |
| 6 | 5.7 | 22 | 14 | **0.5** | 1.1 | 0.8 | 51 | 31 | **5.9** | 16 | 8.9 | 151 | 240 |
| 7 | **2.9** | **7.0** | 16 | **0.4** | 1.0 | 0.6 | 17 | 36 | <LOD | <LOD | **5.8** | 84 | 111 |
| 8 | <LOD | 34 | 29 | 1.2 | 1.9 | 1.1 | 66 | 46 | 10 | 7.5 | 13 | 210 | 312 |
| 9 |  |  |  |  |  |  |  |  |  |  |  |  |  |
| 10 | **2.7** | 357 | 216 | 1.5 | 2.1 | 1.4 | 452 | 293 | <LOD | 22 | 10 | 1355 | 1396 |
| 11 | 220 | 34 | 30 | 1.8 | 3.0 | 1.8 | 96 | 61 | <LOD | 26 | 28 | 282 | 745 |
| 12 | 3.2 | 42 | 44 | **0.4** | **0.7** | 0.4 | 93 | 92 | 9.2 | 18 | 15 | 315 | 427 |
| 13 | <LOD | 33 | 20 | <LOD | <LOD | <LOD | 59 | 23 | <LOD | <LOD | <LOD | 135 | 161 |
| 14 |  |  |  |  |  |  |  |  |  |  |  |  |  |
| 15 | **1.5** | 85 | 86 | <LOD | <LOD | <LOD | 1291 | 918 | <LOD | <LOD | <LOD | 2380 | 2415 |
| 16 | **2.2** | 16 | 10 | <LOD | <LOD | <LOD | 18 | 15 | <LOD | <LOD | <LOD | 59 | 78 |
| 17 | **1.8** | **5.2** | **4.0** | <LOD | <LOD | **0.2** | 6.6 | 5.8 | <LOD | **2.9** | <LOD | 25 | 50 |
| n>LOD | 77% | 100% | 100% | 54% | 54% | 69% | 100% | 100% | 31% | 69% | 54% |  |  |
| n>LOQ | 31% | 77% | 85% | 31% | 46% | 54% | 100% | 100% | 23% | 46% | 46% |  |  |
| Mean | 19 | 64 | 48 | 0.7 | 1.1 | 0.6 | 187 | 135 | 2.8 | 10 | 9.9 | 458 | 545 |
| Median | 2.6 | 33 | 20 | 0.4 | 0.7 | 0.4 | 59 | 36 | 0.0 | 4.4 | 5.8 | 182 | 274 |
| Min | <LOD | <LOQ | <LOQ | <LOD | <LOD | <LOD | 6.6 | 5.8 | <LOD | <LOD | <LOD | 25 | 50 |
| Max | 220 | 357 | 216 | 3.0 | 4.7 | 1.8 | 1291 | 918 | 11 | 37 | 48 | 2380 | 2415 |
| SD | 60 | 99 | 63 | 0.9 | 1.5 | 0.6 | 353 | 250 | 4.5 | 12 | 14 | 685 | 680 |

^1^ Sum of organophosphorus PFASs, not including 6:2 FTSA. ^2^ Sum of all analyzed PFASs.

## Table S 24. PFCA and PFCA concentrations (ng/g dust) determined in dust samples from the extra rooms.

| **Family** | **PFBA** | **PFPeA** | **PFHxA** | **PFHpA** | **PFOA** | **PFNA** | **PFDA** | **PFUnDA** | **PFDoA** | **PFTrA** | **PFTeA** | **PFBS** | **PFHxS** | **PFOS** | **ΣPFCAs/SAs** |
| --- | --- | --- | --- | --- | --- | --- | --- | --- | --- | --- | --- | --- | --- | --- | --- |
| 1 |  |  |  |  |  |  |  |  |  |  |  |  |  |  |  |
| 2 |  |  |  |  |  |  |  |  |  |  |  |  |  |  |  |
| 3 |  |  |  |  |  |  |  |  |  |  |  |  |  |  |  |
| 4 |  |  |  |  |  |  |  |  |  |  |  |  |  |  |  |
| 5 | <LOD | **8.6** | 52 | 9.4 | 27 | 9.4 | 16 | 3.8 | 12 | <LOD | <LOD | 8.7 | 8.1 | 26 | 181 |
| 6 | **21** | **11** | 38 | 16 | 650 | 16 | 35 | 4.4 | 26 | **2.6** | 20 | <LOD | <LOD | 4.4 | 844 |
| 7 |  |  |  |  |  |  |  |  |  |  |  |  |  |  |  |
| 8 |  |  |  |  |  |  |  |  |  |  |  |  |  |  |  |
| 9 |  |  |  |  |  |  |  |  |  |  |  |  |  |  |  |
| 10 | <LOD | <LOD | 17 | <LOD | 5.7 | **1.3** | **1.7** | <LOD | <LOD | <LOD | <LOD | <LOD | **1.3** | 8 | 35 |
| 11 | **18** | <LOD | 33 | 20 | 81 | 9.1 | 9.4 | **2.9** | **5.1** | **2.8** | **4.1** | <LOD | <LOD | 63 | 248 |
| 12 |  |  |  |  |  |  |  |  |  |  |  |  |  |  |  |
| 13 | <LOD | <LOD | **2.9** | <LOD | 4.8 | **2.5** | **1.4** | <LOD | <LOD | <LOD | <LOD | <LOD | <LOD | 41 | 53 |
| 14 |  |  |  |  |  |  |  |  |  |  |  |  |  |  |  |
| 15 |  |  |  |  |  |  |  |  |  |  |  |  |  |  |  |
| 16 |  |  |  |  |  |  |  |  |  |  |  |  |  |  |  |
| 17 |  |  |  |  |  |  |  |  |  |  |  |  |  |  |  |
| n>LOD | 40% | 40% | 100% | 60% | 100% | 100% | 100% | 60% | 60% | 40% | 40% | 20% | 40% | 100% |  |
| n>LOQ | 0% | 0% | 80% | 60% | 100% | 60% | 60% | 40% | 40% | 0% | 20% | 20% | 20% | 100% |  |
| Mean | 7.8 | 3.9 | 29 | 9.1 | 154 | 7.7 | 13 | 2.2 | 8.6 | 1.1 | 4.8 | 1.7 | 1.9 | 28 | 272 |
| Median | 0.0 | 0.0 | 33 | 9.4 | 27 | 9.1 | 9.4 | 2.9 | 5.1 | 0.0 | 0.0 | 0.0 | 0.0 | 26 | 181 |
| Min | <LOD | <LOD | <LOQ | <LOD | 4.8 | <LOQ | <LOQ | <LOD | <LOD | <LOD | <LOD | <LOD | <LOD | 4.4 | 35 |
| Max | 21 | 11 | 52 | 20 | 650 | 16 | 35 | 4.4 | 26 | 2.8 | 20 | 8.7 | 8.1 | 63 | 844 |
| SD | 11 | 5.4 | 19 | 9.1 | 279 | 6.0 | 14 | 2.1 | 11 | 1.5 | 8.7 | 3.9 | 3.5 | 24 | 332 |

## Table S 25. FTSA and organophosphorus PFAS concentrations (ng/g dust) determined in dust samples from the extra rooms.

| **Family** | **6:2 FTSA** | **6:2PAP** | **8:2PAP** | **6:6PFPIA** | **6:8PFPIA** | **8:8PFPIA** | **6:2diPAP** | **8:2diPAP** | **PFHxPA** | **PFOPA** | **PFDPA** | **ΣOP-PFASs^1^** | **ΣPFASs^2^** |
| --- | --- | --- | --- | --- | --- | --- | --- | --- | --- | --- | --- | --- | --- |
| 1 |  |  |  |  |  |  |  |  |  |  |  |  |  |
| 2 |  |  |  |  |  |  |  |  |  |  |  |  |  |
| 3 |  |  |  |  |  |  |  |  |  |  |  |  |  |
| 4 |  |  |  |  |  |  |  |  |  |  |  |  |  |
| 5 | <LOD | **6.2** | **4.3** | <LOD | <LOD | <LOD | 24 | 8.8 | <LOD | <LOD | <LOD | 43 | 224 |
| 6 | 9.1 | 36 | 37 | <LOD | <LOD | **0.1** | 136 | 165 | <LOD | **4.3** | <LOD | 378 | 1232 |
| 7 |  |  |  |  |  |  |  |  |  |  |  |  |  |
| 8 |  |  |  |  |  |  |  |  |  |  |  |  |  |
| 9 |  |  |  |  |  |  |  |  |  |  |  |  |  |
| 10 | **2.6** | 146 | 66 | 1.3 | 2.8 | 1 | 216 | 99 | <LOD | **3.9** | <LOD | 536 | 574 |
| 11 | 26 | 39 | 28 | <LOD | **0.5** | **0.3** | 161 | 78 | <LOD | 4.9 | <LOD | 312 | 586 |
| 12 |  |  |  |  |  |  |  |  |  |  |  |  |  |
| 13 | <LOD | 10 | 6.8 | <LOD | <LOD | <LOD | 33 | 13 | <LOD | <LOD | <LOD | 63 | 115 |
| 14 |  |  |  |  |  |  |  |  |  |  |  |  |  |
| 15 |  |  |  |  |  |  |  |  |  |  |  |  |  |
| 16 |  |  |  |  |  |  |  |  |  |  |  |  |  |
| 17 |  |  |  |  |  |  |  |  |  |  |  |  |  |
| n>LOD | 60% | 100% | 100% | 20% | 40% | 60% | 100% | 100% | 0% | 60% | 0% |  |  |
| n>LOQ | 40% | 80% | 80% | 20% | 20% | 20% | 100% | 100% | 0% | 20% | 0% |  |  |
| Mean | 7.5 | 47 | 28 | 0.3 | 0.7 | 0.3 | 114 | 73 |  | 2.6 |  | 266 | 546 |
| Median | 2.6 | 36 | 28 | 0.0 | 0.0 | 0.1 | 136 | 78 |  | 3.9 |  | 312 | 574 |
| Min | <LOD | <LOQ | <LOQ | <LOD | <LOD | <LOD | 24 | 8.8 |  | <LOD |  | 43 | 115 |
| Max | 26 | 146 | 66 | 1.3 | 2.8 | 1.0 | 216 | 165 |  | 4.9 |  | 536 | 1232 |
| SD | 11 | 57 | 25 | 0.6 | 1.2 | 0.4 | 83 | 65 |  | 2.4 |  | 211 | 436 |

^1^ Sum of organophosphorus PFASs, not including 6:2 FTSA. ^2^ Sum of all analyzed PFASs.

## Figure S 2. Wilcoxon Signed Rank test (paired). Ordered after level of significance, significant differences to the left of the vertical line (p<0.05). Mean values above 1 implies higher values in children's room.

## Figure S 3. Wilcoxon Signed Rank test (paired). Ordered after level of significance, significant differences to the left of the vertical line (p<0.05). Mean values above 1 implies higher values in children's room.

## Figure S 4. Wilcoxon Signed Rank test (paired). Ordered after level of significance, significant differences to the left of the vertical line (p<0.05). Mean values above 1 implies higher values in adult’s bedroom.

## Figure S 5. Principal component analysis (PCA) on the PFAS profile in dust among various rooms. The larger dots represent a center point and the ellipse a confidence interval in which 95% of the dots are expected to fall within. As the centers of gravity overlap, there is no significant difference among type of rooms.

## Figure S 6. Serum (pmol/mL) vs dust (pmol/g) concentrations of perfluoroalkyl carboxylic (PFHpA, PFOA, PFNA, PFDA, PFUnDA) and perfluorooctane sulfonic acid (PFOS) presented on a logarithmic scale. Spearman's rank correlation (one-tailed tests) is showed (r_s_).

## Figure S 7. Serum (pmol/mL) vs dust (pmol/g) concentrations of 6:2 and 8:2 diPAPs. Spearman's rank correlation (one-tailed tests) is showed (r_s_).

## Figure S 8. Logged concentrations of serum-T4 (nmol/L), TSH (µg/L) and the ratio T4/TSH vs PFAS total T4 equivalents (pmol T4 eq/mL). No significant correlations were found, using Spearman's rank correlation (r_s_), two-tailed tests.

## Figure S 9. Significant correlations found for unlogged PFHpA and logged PFUnDA and PFDoDA concentrations vs. serum cholesterol levels, using Spearman's rank correlation (r_s_), one-sided tests.
